# Quality Matters: Systematic Analysis of Endpoints Related to “Cellular Life” in Vitro Data of Radiofrequency Electromagnetic Field Exposure

**DOI:** 10.3390/ijerph13070701

**Published:** 2016-07-12

**Authors:** Myrtill Simkó, Daniel Remondini, Olga Zeni, Maria Rosaria Scarfi

**Affiliations:** 1 Environmental Resources and Technologies, Department Health and Environment, AIT Austrian Institute of Technology, Tulln 3430, Austria; 2 Dipartimento di Fisica e Astronomia, Università di Bologna, Bologna 40126, Italy; daniel.remondini@unibo.it; 3 CNR-Institute for Electromagnetic Sensing of the Environment, Napoli 80124, Italy; zeni.o@irea.cnr.it (O.Z.); scarfi.mr@irea.cnr.it (M.R.S.)

**Keywords:** apoptosis, cell proliferation, association analysis, electromagnetic fields, in vitro

## Abstract

Possible hazardous effects of radiofrequency electromagnetic fields (RF-EMF) at low exposure levels are controversially discussed due to inconsistent study findings. Therefore, the main focus of the present study is to detect if any statistical association exists between RF-EMF and cellular responses, considering cell proliferation and apoptosis endpoints separately and with both combined as a group of “cellular life” to increase the statistical power of the analysis. We searched for publications regarding RF-EMF in vitro studies in the PubMed database for the period 1995–2014 and extracted the data to the relevant parameters, such as cell culture type, frequency, exposure duration, SAR, and five exposure-related quality criteria. These parameters were used for an association study with the experimental outcome in terms of the defined endpoints. We identified 104 published articles, from which 483 different experiments were extracted and analyzed. Cellular responses after exposure to RF-EMF were significantly associated to cell lines rather than to primary cells. No other experimental parameter was significantly associated with cellular responses. A highly significant negative association with exposure condition-quality and cellular responses was detected, showing that the more the quality criteria requirements were satisfied, the smaller the number of detected cellular responses. According to our knowledge, this is the first systematic analysis of specific RF-EMF bio-effects in association to exposure quality, highlighting the need for more stringent quality procedures for the exposure conditions.

## 1. Introduction

There are many national and international organizations, committees, and institutions performing health risk assessment regarding exposure to radiofrequency electromagnetic fields (RF-EMF) with various outcomes. Among them are the World Health Organization (WHO), the International Commission on Non-Ionizing Radiation Protection (ICNIRP), the International Agency for Research on Cancer (IARC), the Institute of Electrical and Electronic Engineers (IEEE), and the European Commission (SCENIHR, Scientific Committee for the Emerging and Newly Identified Health Risks). In a recent review [1], the different evaluations were summarized and analyzed with the general conclusion that more research is needed to get answers to health risk-related questions.

There is a vast amount of scientific literature regarding the biological effects of RF-EMF in in vitro and in vivo investigations, but many epidemiological studies have also been carried out with inconsistent results. In addition, many review articles have summarized the state of the art in almost all areas of biology, considering different living systems, exposure levels (specific absorption rates, SAR), frequencies, exposure duration, and so on (for some of the recent review articles, see [1,2,3,4,5,6,7,8]). The main concern regards possible biological effects at the non-thermal level of RF exposure. This is of fundamental relevance for everyday low-power applications of RF, such as cell phones, Wi-Fi connections, etc., which operate at power levels where tissue-heating (thermal) effects are not expected to occur. Although experimental studies have been performed for decades, there is still controversy on possible effects at these low exposure levels, especially since there is no convincing mechanistic explanation of a biological effect. In addition, there are no consistent findings supporting any adverse effects.

In vitro studies are the most common for the evaluation of biological effects of RF-EMF. They are carried out on tissue or cell cultures of animal or human origin, on transformed or primary cells and cell lines. Moreover, they are relatively simple to handle and represent well-described in models, where the control of experimental conditions, including exposure, is significantly better than in vivo animal or human studies.

Cell proliferation and apoptosis—in vitro processes that are studied for cellular responses of RF-EMF exposure—are among the most investigated. They are fundamental processes in multicellular organisms and are tightly connected to each other. Cell proliferation is an increase in cell number, and may result from increased growth and division or from decreased cell death. Cell proliferation can be stimulated by physiological and pathological conditions and is largely controlled (stimulated or inhibited) by signals from the microenvironment. An excess of stimulators or a deficiency of inhibitors leads to net growth and, in the case of cancer, uncontrolled growth.

Apoptosis is a physiological process essential for balanced tissue homeostasis and is thus part of cell growth. There are several lines of evidence linking apoptosis to proliferation (see [9]). Nevertheless, apoptosis can also be induced by a variety of pathological stimuli. Apoptosis can be triggered by intracellular or extracellular signals which follow two main pathways: the intrinsic or mitochondrial pathway, which transmits intracellular received death signals, and the extrinsic or death receptor pathway, relaying apoptotic messages via receptors.

Here we chose to analyze investigations related to cell proliferation and apoptosis, considered both separately and combined into a group of “cellular life”. The main goal of this study is the use of the “grouping approach” to experimental parameters [10], and thus to test if any statistical association exists between RF-EMF and cellular responses. Furthermore, we define five criteria to analyze the quality of the exposure conditions within the selected studies. We think that this approach enables a more comprehensive analysis of available data to detect an association between cellular response(s) and RF-EMF exposure parameters. Moreover, this data evaluation allows an independent and unbiased execution of the analysis, which can contribute to a better understanding of RF-EMF exposure and cellular response(s).

## 2. Materials and Methods 

### 2.1. Literature Search

A literature search was conducted by using the PubMed (www.ncbi.nlm.nih.gov/pubmed) bibliographic database. Keywords used for the exposure element of the studies were: electromagnetic fields, microwaves, radio waves, nonionizing, radiofrequency, cellular phone, mobile phone, base station, GSM, UMTS, and mobile communication. For the outcome elements, the following key words were used: apoptosis, programmed cell death, annexin, tunel, phosphatidylserine, mitochondrial pathway, death receptor, chromatin condensation, caspase, caspase cleavage, cell cycle, cell cycle progression, cyclins, and cell proliferation. Peer reviewed original articles from 1995 to 2014 in the English language were considered, whereas review articles were disregarded.

All identified investigations, with the exception of whole genomic or proteomic studies, were included in the analysis. Consequently, no exclusion criteria (regarding exposure conditions) were applied. Since in several cases a paper reported more than one tested experimental condition (e.g., due to a different exposure duration or SAR), all different experimental results were extracted and analyzed.

### 2.2. Data Extraction

A spread-sheet document was built up to assist data extraction from all the experiments recognized. For each experiment we recorded the following information: bibliographic reference, cell culture type (considering primary cells and cell lines, in the following referred to as “cell type”), investigated endpoints (apoptosis or cell proliferation), outcome (cellular response/no response, whereby “response” is referred to if the paper reported a statistically significant (*p* < 0.05) outcome in comparison to the respective control, independently of the direction), exposure frequency, duration, and SAR value. Furthermore, we collected data about frequency modulation, co-exposure(s), and statistical power. As “quality criteria” we defined the presence of sham-exposure, appropriate dosimetry, use of positive control, blinded analysis, and temperature control.

### 2.3. Data Analysis 

First, we performed descriptive statistics of the selected parameters in order to characterize the publications both over time (i.e., in the period from 1995 to 2014) and by cell type (primary cells or cell lines). Secondly, we tested the hypothesis that the outcomes of the experiments could be significantly associated to specific factors such as biological endpoint, cell type, exposure frequency, exposure duration, and SAR. Regarding biological endpoints, apoptosis, cell proliferation, and both combined as a group of “cellular life” were considered, while cell type was categorized as primary cells and cell lines. For the frequency variable, the intervals considered were: 0.5–1 GHz, 1–3 GHz, 3–10 GHz, and >10 GHz. These intervals were chosen in accordance with the frequency intervals of the most common RF-EMF appliances.

Intervals for the exposure duration were chosen as acute (≤60 min), long (1–24 h), and chronic (day/s) exposure, whereby the latter group includes those experiments that used intermittent exposure over several days.

Finally, the SAR values were pooled into three groups (≤1 W/kg, 1–2 W/kg, >2 W/kg) with regard to under, around, and above the safety limits [6]. The variables and group ranges are reported in Table 1. Furthermore, the quality of the performed studies was also taken into account. We considered the highest quality criteria (labelled as Q5) when an experiment satisfied all five quality controls: sham, dosimetry control, temperature control, blinded manner, and positive control. Experiments labelled Q4 to Q1 refer to those in which four to one quality criteria were satisfied. We did not rank these criteria. However, we also tested the specific association of the experimental outcome with sham and dosimetry, or sham, dosimetry, and temperature, as specific cases of Q2 and Q3, respectively, since these criteria are often mentioned in the literature as the most relevant, and also because these were the most present criteria [11,12,13,14,15].

We performed a Fisher exact test (Mathworks Matlab software) for the association of each selected variable with the experimental outcome (no response/response). In cases in which the variable was classified into more than two intervals (e.g., exposure frequency), each interval was tested for association with the outcome in comparison with all the remaining groups. Furthermore, for the quality criteria variables, we performed an association test for each variable—namely, the presence of a minimum number (from 1 to 5) of satisfied quality criteria against the experiments with a smaller number of satisfied criteria. We defined a significance threshold *p* = 0.05 for our analyses.

## 3. Results

We identified 104 peer-reviewed publications, and extracted 483 different experimental results dealing with apoptosis or cell cycle/proliferation (Table 2 and Supplementary Material Table S1 including all information). All responses were considered separately, independently of the size or direction of the response but considering the given *p* < 0.05, as described in the related paper. We did not consider whole genomic or proteomic studies, since in these cases the responses are too complex to be condensed in a response/no response dichotomy.

**Figure 1:**
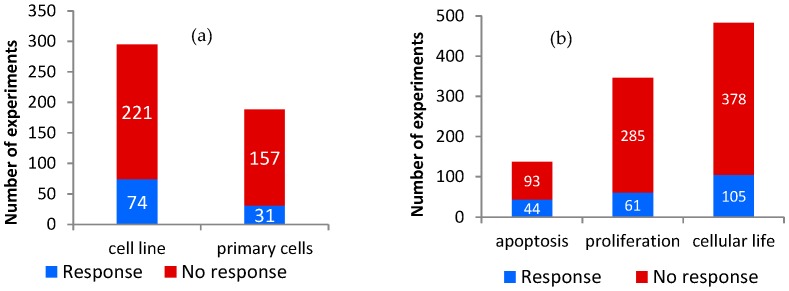
Cellular response in the different groups considered in the analysis: Number of experiments resulting in response or no response for (**a**) “cellular life” endpoint stratified by cell type; and (**b**) apoptosis and proliferation separately and combined in “cellular life” endpoint.

The identified experiments were carried out on 28 different primary cell types (188 experiments) and 46 different cell lines (295 experiments) (Figure 1). The “cellular life” group resulted in 105 cellular responses out of 483 total experiments (22%). The Fisher test detected a significant correlation between cellular responses on “cellular life” and cell lines, as compared to primary cells (*p* = 0.016). In other words, it is more likely that cellular responses regarding this outcome appear in cell lines than in primary cells (Figure 1a).

Apoptosis was affected in 44 cases among 137 (32%), while proliferation was affected in 61 experiments among 346 (18%), with a significant association of responses to apoptosis versus proliferation (*p* = 0.0005, Figure 1b).

No association between any biological endpoint and frequency group was detected (0.5–1 GHz, 214 experiments; 1–3 GHz, 237 experiments; 3–10 GHz, 13 experiments; >10 GHz, 19 experiments). We noticed 12 different modulations within the performed experiments. However, because of the small number of experiments for each modulation scheme, the analysis showed a very weak statistical power. Thus, this part of the analysis was not feasible.

**Figure 2:**
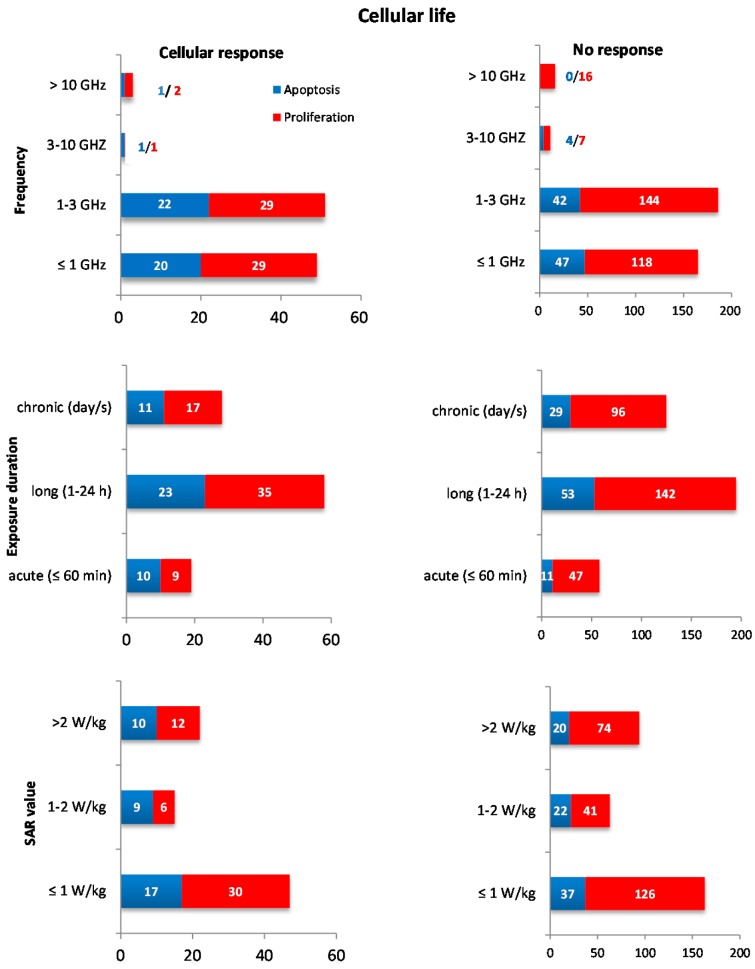
Compilation of data: The bar charts describe the number of experiments reporting cellular responses (left panel) and no responses (right panel) for “cellular life”, stratified by apoptosis (blue) and cell proliferation (red) endpoints, and stratified into specific groups for frequency, exposure duration, and SAR value.

Exposure duration was grouped as acute (≤60 min, 77 experiments), long (1–24 h, 253 experiments) and chronic (>24 h, 153 experiments). No correlation between exposure duration and cellular response was detected. The association test with SAR level was carried out on experiments in which the SAR value was given in the publication; thus, only 404 experiments were considered (210 for SAR < 1 W/kg; 78 for 1–2 W/kg; and 116 for SAR > 2 W/kg). In addition, in this case, no association was detected (all association tests for frequency, exposure duration, and SAR intervals are listed in the Supplementary Material Table S2). An overview of the data extracted from all experiments is given in Figure 2, where the outcomes are reported for all groups considered.

**Figure 3:**
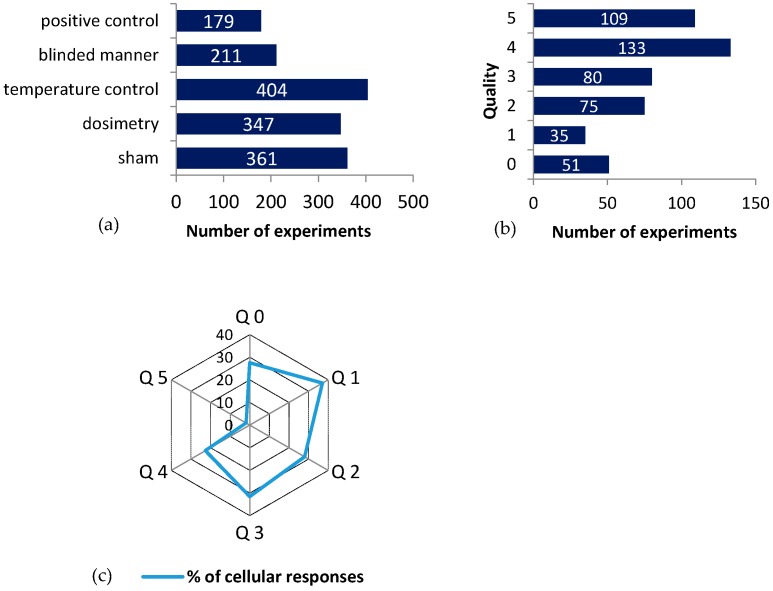
The quality of data: (**a**) The bar chart of the experiments that satisfied the listed quality criteria (y axis); (**b**) Experiments that satisfied a given number of quality criteria out of a total of 483 experiments; (**c**) Spider net plot of the percentage of cellular responses as a function of the quality of the experiments. Quality ranges from 0 (no criteria satisfied, Q0) to 5 (all criteria satisfied, Q5).

The exposure quality was evaluated for all experiments in association to the “cellular life” group, according to the five selected criteria: sham (RF-control without RF exposure), dosimetry control, temperature control, blinded manner, and positive control. Sham exposure was used in 361 experiments, 347 experiments included numerical and/or experimental dosimetry, 404 included temperature control, 211 were performed in a blinded manner, and 179 included positive controls (Figure 3). There were 51 experiments using none of the mentioned quality criteria (Q0, 11%), about 23% (109 experiments) satisfied all the quality criteria, and about 67% (323 experiments) satisfy from one to four criteria (Figure 3).

The ratio of cellular responses to RF-EMF exposure is between 22% and 37% in all experiments, in which no or parts of the quality criteria are satisfied (Q0–Q4, see Figure 3c). The Q5 group has less than 2% cellular responses (2 experiments out of 109). Fisher test for the Q5 group showed a strongly significant association between no responses and high-quality exposure conditions (*p* = 10^−10^).

In addition, the other association studies related to quality (i.e., the Q1–Q4 cases, the Sham+Dosimetry, or the Sham + Dosimetry + Temperature cases) produced similar results—namely, in all cases, a smaller number of cellular responses was found for higher-quality experiments (*p* < 10^−5^, see Table 3). The data show that the lower the quality criteria of the exposure condition, the more cellular responses are detected, with a large drop in responses for experiments in which all quality criteria are satisfied.

**Figure 4:**
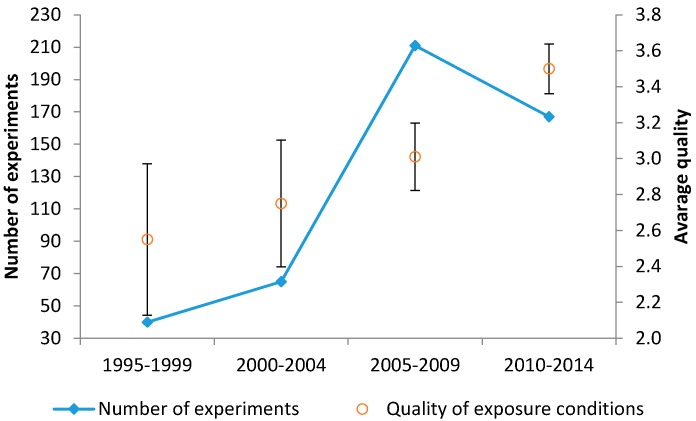
Average quality level of the experiments over the years: Number of experiments from 1995 to 2014 (with five-year intervals) (left y-axis) in parallel to the average quality score of the experiments (right y-axis).

As a descriptive analysis, the trend of the average exposure quality level over time for the considered experiments is presented in Figure 4. It appears that between 2005 and 2009, the overall number of experiments peaked (perhaps related to the scientific and public concern about cell-phone usage that was rapidly expanding in those years), with an average quality of around Q3 (three quality criteria satisfied). The number of studies has declined over the last five years of our survey period, accompanied by a small but significant increase of the average quality criteria to 3.5. A significant difference between the average quality of the studies before and after 2005 was detected (*p* = 10^−5^).

## 4. Discussion

The possible effect of exposure to non-thermal RF-EMF, where the exposure is not causing any tissue heating, causes controversies. A common concern is about the possible long-term health effects of exposure at very low (below tissue heating) levels. In 2013, an expert group of the International Agency for Research on Cancer [5] classified RF-EMF as a possible carcinogen for humans (risk group 2B) since there is “limited evidence” for the carcinogenicity, based on positive associations in epidemiological studies between glioma and acoustic neuroma and exposure to RF-EMF. More recently, epidemiological studies have not confirmed any correlation between RF-EMF exposure and disease development (for a review see [8]). Furthermore, there are no experimental findings that can provide a mechanistic explanation for such an outcome, thus no established biological or biophysical mechanism of action exists so far.

The majority of the studies regarding effects of RF-exposure available in the literature refer to *in vitro* investigations, due to their key role in advancing knowledge about the possible relationship between RF exposures and human diseases. These studies display inconsistent results, possibly due to differences in the used cell type, frequency, SAR value, exposure duration, and observed cellular endpoint(s). Thus, the question of whether non-thermal RF exposure can alter cellular function in vitro still remains unresolved. Therefore, in the present study, we aimed to analyze specific in vitro data by using a statistical non-biased method to see if there is any association between positive findings and RF-EMF exposure. We chose the period from 1995 to 2014 since in this period the overall use of mobile telephones and wireless communication dramatically increased worldwide. In particular, we focused on apoptosis and cell proliferation, which are two major cellular processes that have been frequently investigated in RF-EMF related in vitro studies. Here we addressed all available published data in cellular and molecular investigations without applying any exclusion criteria, but not considering whole proteomic and genomic studies. Both endpoints were analyzed separately, and also combined in a group of “cellular life”, improving the statistical power of the analysis. For the same reason, the extracted experiments were pooled into specific groups and intervals (cell type, frequency, exposure duration, and SAR value) in order to see if there is any statistical association of cellular responses with one or more of these categories. For our purposes, the statistically significant (*p* < 0.05) biological effect observed in the related publication, independent of the direction of the outcome, was considered as a cellular response.

Our analysis showed both cellular responses and no responses, where cellular responses were significantly associated with apoptosis rather than with cell proliferation, with more experiments focusing on proliferation (346) than on apoptosis (137). We also detected a higher association of cellular responses with cell lines (295) rather than with primary cells (188). At the present stage, we have no explanation for this association. There seems to be an imbalance in the numbers of specific investigations; however, the outcome of the test should not be affected by that.

The grouped experiments for frequency, exposure duration, and SAR values did not produce any statistical association with cellular responses including high SAR values (>2 W/kg) and chronic exposure duration (≥24 h); however, according to experiments from “classical” toxicological studies, one could otherwise expect that higher exposure levels and longer exposure duration would foster cellular responses.

Data were not grouped and analyzed for modulation scheme, since no associations between frequency and cellular responses were detected. Moreover, the scanty number of experiments for each of the twelve modulation schemes identified would have led to unreliable results due to the weak statistical power.

*In vitro* experiments which aim to study cellular response(s) of RF-EMF exposure need specific experimental controls to reduce confounding variables. These variables could otherwise affect the results and impair reliability and reproducibility of the experiments. On the basis of the suggestions from the World Health Organization (WHO) in the EMF Project [120] and of published general guidelines for RF in vitro exposure setups [11,12,13,14,15], we considered five relevant quality criteria for RF exposure of cell cultures (temperature control, dosimetry, sham, positive control, and blinded experimental condition). We want to stress that these criteria were not ranked, since we considered all of them equally relevant for good laboratory practices. In detail, since it is known that certain RF-EMF setups induce a temperature increase within the exposed cell culture dishes, and thus may cause false positive cellular responses, temperature must be monitored by using non-perturbing probes and active cooling with either forced air or water. SAR (W/kg) is a measure of the absorbed energy rate by the human body/tissue exposed to RF-EMF, and is widely used for the international regulation of protection against electromagnetic fields. Furthermore, it is suitable to use this parameter to compare biological effects observed under different exposure conditions. Monitoring the SAR distribution pattern by means of numerical and/or empirical analysis within the cell cultures, is of paramount importance and allows the avoidance of undesired “hot spots” that can cause local heating within the sample. The sham control sample (cell culture placed in a RF exposure device identical to the one employed for the exposure but with zero RF) represents the true control, taking into account the microenvironment in the exposure device and the experimental conditions that could affect the cellular endpoint under examination. Positive controls provide evidence for controlled experimental conditions, assuring that the assay methodology is responding adequately to a well-known agent. Moreover, performing experiments in a blind manner, with samples coded so that their treatment group is unknown until the data are analyzed, will allow for the prevention of any kind of bias in data analysis. This is particularly relevant, especially when a slight variation is expected, as it is for RF exposure.

By introducing the five above-mentioned quality criteria, all identified cellular responses were tested for association, grouped as (Q0), Q1 to Q5 according to the number of satisfied criteria. Interestingly, the lower the quality of the exposure condition, the more responses were detected. This was true even when three specific exposure criteria were considered—namely, temperature, sham exposure, and dosimetry. In particular, when moving from Q1 to Q5, we observed an increasing negative association of the responses and number of satisfied criteria, with a significant drop in cellular responses for Q5. When all quality criteria were satisfied, only two experiments of 109 showed cellular responses, against an average response of about 27% for the Q1–Q4 cases. These findings further highlight the importance of performing experiments under strictly-controlled exposure conditions, in order to clarify the conflicting results reported in the literature. Only 109 experiments out of 483 (<25%) in the period of 1995–2014 satisfied the highest quality denominator Q5. Here we have observed that, when comparing the literature from the last five years with the preceding period, the average quality has significantly increased from 3 to 3.5 (Figure 4) with 105 Q5 records in the most recent decade, but it is still very far from an optimal control of experimental procedures (they correspond to less than 30% of the total record of the decade).

## 5. Conclusions

Taking it together, our analysis shows that in vitro cellular response after exposure to RF-EMF—Considering apoptosis and cell proliferation separately and both combined in a group of “cellular life”—have no significant association to any of the relevant exposure parameters, such as frequency, exposure duration, or SAR value. Only cell lines showed a statistically significant higher association with cellular response, as compared to primary cell cultures. The most relevant result in the present study is the negative association between outcomes of cellular responses and the quality of the experimental procedures, specifically to the exposure conditions. The more the quality criteria requirements were satisfied, the smaller the number of cellular responses that were detected, with a dramatic drop in the number of responses at the highest possible quality criteria number (<2% in more than 100 experiments).

Our study provides evidence that the accurate control of the exposure and experimental procedures is crucial. Therefore, we suggest the definition of an appropriate Standard Operating Procedure (SOP) for EMF research (at least for the investigation of non-thermal RF-EMF effects) or a rigorous definition of a unified “best practice”, such as the MIAME procedure for gene expression microarray experiments [121].

We think that our analysis provides robust results investigating the association of several RF-EMF experimental conditions to selected biological endpoints. Moreover, the results shows that improving the experimental quality by means of appropriate procedural protocols might allow addressing controversies in EMF research more clearly.

## Figures and Tables

**Table 1:** Variables and group ranges of selected parameters.

Biological Endpoint	Cell Type	Frequency	Exposure Duration	SAR
apoptosis	primary cells	0.5–1 GHz	acute (≤60 min)	≤1 W/kg
cell proliferation	cell lines	1–3 GHz	long (1–24 h)	1–2 W/kg
“cellular life”		3–10 GHZ	chronic (day/s)	>2 W/kg
		>10 GHz		

SAR: Specific Absorption Rate.

**Table 2:** Identified publications and number of experiments.

Reference	Number of Experiments	Reference	Number of Experiments	Reference	Number of Experiments	Reference	Number of Experiments
[16]	15	[17]	2	[18]	4	[19]	4
[20]	18	[21]	2	[22]	3	[23]	2
[24]	2	[25]	3	[26]	4	[27]	2
[28]	4	[29]	6	[30]	2	[31]	6
[32]	1	[33]	2	[34]	4	[35]	1
[36]	1	[37]	8	[38]	3	[39]	2
[40]	1	[41]	2	[42]	4	[43]	1
[44]	1	[45]	4	[46]	2	[47]	7
[48]	2	[49]	1	[50]	2	[51]	10
[52]	2	[53]	3	[54]	4	[55]	2
[56]	2	[57]	1	[58]	6	[59]	1
[60]	4	[61]	2	[62]	9	[63]	7
[64]	2	[65]	3	[66]	10	[67]	4
[68]	1	[69]	3	[70]	2	[71]	2
[72]	11	[73]	1	[74]	36	[75]	7
[76]	1	[77]	12	[78]	4	[79]	13
[80]	1	[81]	12	[82]	3	[83]	10
[84]	4	[85]	5	[86]	2	[87]	2
[88]	1	[89]	2	[90]	4	[91]	2
[92]	1	[93]	2	[94]	10	[95]	2
[96]	2	[97]	6	[98]	2	[99]	2
[100]	2	[101]	4	[102]	8	[103]	1
[104]	2	[105]	4	[106]	3	[107]	3
[108]	4	[109]	4	[110]	1	[111]	14
[112]	8	[113]	12	[114]	12	[115]	16
[116]	3	[117]	8	[118]	1	[119]	5

**Table 3:** Statistics for the association of quality scores with radiofrequency electromagnetic fields (RF-EMF) response.

Quality Score ^a^	High-Q (%)	Low-Q (%)	*p* Value ^d^
1	21.1	27.5	0.19
2	19.6	31.4	0.014
3	17.7	29.8	0.002
4	13.2	30.3	4 × 10^−6^
5	1.8	27.5	4 × 10^−11^
S + D ^b^	15.5	34.2	4 × 10^−6^
S + D + T ^c^	16.1	31.8	6 × 10^−5^

^a^ “Quality Score” column defines the range for the high-quality class (the number of criteria to be satisfied); ^b^ “S + D” defines the experiments in which at least the Sham and the Dosimetry quality criteria are satisfied; ^c^ “S + D + T” defines the experiments with at least Sham, Dosimetry, and Temperature criteria are satisfied; ^d^ *p* values describe significant association between cellular response and lower-quality experiments.

## Supplementary Materials

The following are available online at www.mdpi.com/1660-4601/13/7/701/s1, Table S1: List of selected publications and extracted experiments related to apoptosis and cell proliferation after exposure to RF-EMF, Table S2: Association test for exposure frequency, duration and SAR intervals.
