# Supplementary material for: Quality Matters: Systematic Analysis of Endpoints Related to “Cellular Life” in Vitro Data of Radiofrequency Electromagnetic Field Exposure
Source: Int J Environ Res Public Health. 2016 Jul 12;13(7):701. doi: 10.3390/ijerph13070701 (PMC4962242; doi:10.3390/ijerph13070701)
Supplement: Supplementary file 1 [file ijerph-13-00701-s001.pdf]

Supplementary Materials: Quality matters: Systematic Analysis of Endpoints Related to "Cellular Life" in Vitro Data of Radiofrequency Electromagnetic Field Exposure

Myrtill Simkó, Daniel Remondini, Olga Zeni and Maria Rosaria Scarfi

Table S1. List of selected publications and extracted experiments related to apoptosis and cell proliferation after exposure to RF-EMF.

References	Cells	Primary Cell	Cell Line	Frequency	Power Flux Density	SAR (W/Kg)	Time of Exposure	Apoptosis	Response	Proliferation	Response	Donors/ n Numbers	*p* Value	Sham	Dosimetry	Positive Control	Blind	Temperature Control
[1]	Human peripheral blood lymphocytes	x		380 MHz		0.08	48 h			x	no	15	<0.05	no	yes	no	no	yes
	Human peripheral blood lymphocytes	x		900 MHz		0.208	48 h			x	no	15	<0.05	no	yes	no	no	yes
	Human peripheral blood lymphocytes	x		1800 MHz		1.7	48 h			x	no	15	<0.05	no	yes	no	no	yes
	Human peripheral blood lymphocytes	x		380 MHz		0.08	52h			x	no	15	<0.05	no	yes	no	no	yes
	Human peripheral blood lymphocytes	x		900 MHz		0.208	52h			x	no	15	<0.05	no	yes	no	no	yes
	Human peripheral blood lymphocytes	x		1800 MHz		1.7	52h			x	no	15	<0.05	no	yes	no	no	yes
	Human peripheral blood lymphocytes	x		380 MHz		0.08	56 h			x	no	15	<0.05	no	yes	no	no	yes
	Human peripheral blood lymphocytes	x		900 MHz		0.208	56 h			x	no	15	<0.05	no	yes	no	no	yes
	Human peripheral blood lymphocytes	x		1800 MHz		1.7	56 h			x	no	15	<0.05	no	yes	no	no	yes
	Human peripheral blood lymphocytes	x		380 MHz		0.08	64 h			x	no	15	<0.05	no	yes	no	no	yes
	Human peripheral blood lymphocytes	x		900 MHz		0.208	64 h			x	no	15	<0.05	no	yes	no	no	yes
	Human peripheral blood lymphocytes	x		1800 MHz		1.7	64 h			x	no	15	<0.05	no	yes	no	no	yes
	Human peripheral blood lymphocytes	x		380 MHz		0.08	68 h			x	no	15	<0.05	no	yes	no	no	yes
	Human peripheral blood lymphocytes	x		900 MHz		0.208	68 h			x	no	15	<0.05	no	yes	no	no	yes
	Human peripheral blood lymphocytes	x		1800 MHz		1.7	68 h			x	no	15	<0.05	no	yes	no	no	yes
[2]	Human peripheral mononuclear cells	x		450 MHz	no	no	2 h	x	no			5 donors	no	no	no	no	no	no
	Human peripheral mononuclear cells	x		450 MHz	no	no	6 h	x	no			5 donors	no	no	no	no	no	no
	Human peripheral mononuclear cells	x		450 MHz	no	no	24 h	x	no			5 donors	no	no	no	no	no	no
	Human peripheral mononuclear cells	x		900 MHz	no	no	2 h	x	no			5 donors	no	no	no	no	no	no

	Human peripheral mononuclear cells	x		900 MHz	no	no	6 h	x	no			5 donors	no	no	no	no	no	no
	Human peripheral mononuclear cells	x		900 MHz	no	no	24 h	x	no			5 donors	no	no	no	no	no	no
	Human peripheral mononuclear cells	x		1784 MHz	no	no	2 h	x	no			5 donors	no	no	no	no	no	no
	Human peripheral mononuclear cells	x		1784 MHz	no	no	6 h	x	no			5 donors	no	no	no	no	no	no
	Human peripheral mononuclear cells	x		1784 MHz	no	no	24 h	x	no			5 donors	no	no	no	no	no	no
	Human peripheral mononuclear cells	x		450 MHz	no	no	2 h			x	no	5 donors	no	no	no	no	no	no
	Human peripheral mononuclear cells	x		450 MHz	no	no	6 h			x	no	5 donors	no	no	no	no	no	no
	Human peripheral mononuclear cells	x		450 MHz	no	no	24 h			x	no	5 donors	no	no	no	no	no	no
	Human peripheral mononuclear cells	x		900 MHz	no	no	2 h			x	no	5 donors	no	no	no	no	no	no
	Human peripheral mononuclear cells	x		900 MHz	no	no	6 h			x	no	5 donors	no	no	no	no	no	no
	Human peripheral mononuclear cells	x		900 MHz	no	no	24 h			x	no	5 donors	no	no	no	no	no	no
	Human peripheral mononuclear cells	x		1784 MHz	no	no	2 h			x	no	5 donors	no	no	no	no	no	no
	Human peripheral mononuclear cells	x		1784 MHz	no	no	6 h			x	no	5 donors	no	no	no	no	no	no
	Human peripheral mononuclear cells	x		1784 MHz	no	no	24 h			x	no	5 donors	no	no	no	no	no	no
[3]	Spermatozoa	x		2400 MHz	0.7 μW/cm²		4 h	x	no			29 donors	<0.01	no	no	no	no	yes
	Spermatozoa	x		2400 MHz	0.7 μW/cm²		4 h			x	yes	29 donors	<0.01	no	no	no	no	yes
[4]	Human lymphocytes	x		915 MHz		0.037	2 h	x	no			7	no	yes	yes	yes	yes	yes
	Human lymphocytes	x		915 MHz		0.037	2 h			x	no	7	no	yes	yes	yes	yes	yes
[5]	RPMI 7932 cell line (human melanoma cells)		x	65 GHz	0.07 μWatt/cm²		1 h			x	no	n.s.*	<0.05	no	no	no	no	no
	RPMI 7932 cell line (human melanoma cells)		x	65 GHz	0.07 μWatt/cm²		3 h			x	yes	n.s.	<0.05	no	no	no	no	no
	RPMI 7932 cell line (human melanoma cells)		x	51.05 GHz	0.07 μWatt/cm²		1 h			x	no	n.s.	<0.05	no	no	no	no	no
	RPMI 7932 cell line (human melanoma cells)		x	51.05 GHz	0.07 μWatt/cm²		3 h			x	no	n.s.	<0.05	no	no	no	no	no
	RPMI 7932 cell line (human melanoma cells)		x	65.00 GHz	0.07 μWatt/cm²		1 h			x	no	n.s.	<0.05	no	no	no	no	no
	RPMI 7932 cell line (human melanoma cells)		x	65.00 GHz	0.07 μWatt/cm²		3 h			x	no	n.s.	<0.05	no	no	no	no	no
[6]	K562 cells (human erythroleukemic cell line)		x	65.00 GHz	less than 1 μW/cm²		Intermittent			x	yes	3	<0.05	yes	no	no	no	no
[7]	RPMI 7932 cell line (human melanoma cells)		x	42.2 GHz	0.11 mW/cm²		Intermittent			x	no	2	<0.05	yes	no	no	no	yes

	RPMI 7932 cell line (human melanoma cells)		x	53.57 GHz	0.26 mW/cm²		Intermittent			x	no	2	<0.05	yes	no	no	no	yes
[8]	Human amniotic Cells	x		900 MHz		0.25	24 h	x	no			3	<0.05	yes	yes	yes	no	yes
[9]	Human amniotic Cells	x		900 MHz		1	24 h	x	no			3	<0.05	yes	yes	yes	no	yes
	Human amniotic Cells	x		900 MHz		2	24 h	x	no			3	<0.05	yes	yes	yes	no	yes
	Human amniotic Cells	x		900 MHz		4	24 h	x	no			3	<0.05	yes	yes	yes	no	yes
	Human amniotic Cells	x		900 MHz		0.25	24 h			x	no	3	<0.05	yes	yes	yes	no	yes
	Human amniotic Cells	x		900 MHz		1	24 h			x	no	3	<0.05	yes	yes	yes	no	yes
	Human amniotic Cells	x		900 MHz		2	24 h			x	no	3	<0.05	yes	yes	yes	no	yes
	Human amniotic Cells	x		900 MHz		4	24 h			x	no	3	<0.05	yes	yes	yes	no	yes
[10]	SH-SY5Y (human neuroblastoma cell line)		x	900 MHz		1	5 min	x	yes			3	<0.05; <0.01	yes	yes	no	yes	yes
	SH-SY5Y (human neuroblastoma cell line)		x	900 MHz		1	15 min	x	yes			3	<0.05; <0.01	yes	yes	no	yes	yes
	SH-SY5Y (human neuroblastoma cell line)		x	900 MHz		1	30 min	x	yes			3	<0.05; <0.01	yes	yes	no	yes	yes
	SH-SY5Y (human neuroblastoma cell line)		x	900 MHz		1	6 h	x	yes			3	<0.05; <0.01	yes	yes	no	yes	yes
	SH-SY5Y (human neuroblastoma cell line)		x	900 MHz		1	24 h	x	yes			3	<0.05; <0.01	yes	yes	no	yes	yes
	SH-SY5Y (human neuroblastoma cell line)		x	900 MHz		1	5 min			x	yes	3	<0.05; <0.01	yes	yes	no	yes	yes
	SH-SY5Y (human neuroblastoma cell line)		x	900 MHz		1	15 min			x	yes	3	<0.05; <0.01	yes	yes	no	yes	yes
	SH-SY5Y (human neuroblastoma cell line)		x	900 MHz		1	30 min			x	yes	3	<0.05; <0.01	yes	yes	no	yes	yes
	SH-SY5Y (human neuroblastoma cell line)		x	900 MHz		1	6 h			x	yes	3	<0.05; <0.01	yes	yes	no	yes	yes
	SH-SY5Y (human neuroblastoma cell line)		x	900 MHz		1	24 h			x	yes	3	<0.05; <0.01	yes	yes	no	yes	yes
[11]	SH-SY5Y		x	1800 MHz		0.086	2 h	x	no			4	no	yes	no	no	no	yes
	SH-SY5Y		x	1800 MHz		0.086	4 h	x	no			4	no	yes	no	no	no	yes
[12]	Burkit lymphoma cells (Raji)		x	1800 MHz		0.35	24 h	x	yes			5	<0.05	no	yes	no	no	yes
[13]	SHG44 cells (human glioma cells)		x	900 MHz	2 mW/cm²		Intermittent	x	no			3	<0.05	no	no	yes	no	no

	SHG44 cells (human glioma cells)		x	900 MHz	4 mW/cm²		Intermittent	x	yes			3	<0.05	no	no	yes	no	no
	SHG44 cells (human glioma cells)		x	900 MHz	6 mW/cm²		Intermittent	x	yes			3	<0.05	no	no	yes	no	no
	SHG44 cells (human glioma cells)		x	900 MHz	2mW/cm²		Intermittent			x	no	3	<0.05	no	no	yes	no	no
	SHG44 cells (human glioma cells)		x	900 MHz	4 mW/cm²		Intermittent			x	yes	3	<0.05	no	no	yes	no	no
	SHG44 cells (human glioma cells)		x	900 MHz	6 mW/cm²		Intermittent			x	yes	3	<0.05	no	no	yes	no	no
	Human peripheral blood mononuclear cells	x		1800 MHz		2	44 h	x	no			5-10 donors	<0.05	yes	yes	no	no	yes
[14]	Human peripheral blood mononuclear cells	x		1800 MHz		2	44 h	x	no			5-10 donors	<0.05	yes	yes	no	no	yes
	Human peripheral blood mononuclear cells	x		1800 MHz		1.4	44 h	x	no			5-10 donors	<0.05	yes	yes	no	no	yes
	Human peripheral blood mononuclear cells	x		900 MHz		0.073	Intermittent	x	no			25 donors	<0.05	yes	yes	no	no	yes
	Human peripheral blood mononuclear cells	x		900 MHz		0.073	Intermittent	x	no			25 donors	<0.05	yes	yes	no	no	yes
	Human peripheral blood mononuclear cells	x		1800 MHz		2	44 h			x	no	5-10 donors	<0.05	yes	yes	no	no	yes
	Human peripheral blood mononuclear cells	x		1800 MHz		2	44 h			x	no	5-10 donors	<0.05	yes	yes	no	no	yes
	Human peripheral blood mononuclear cells	x		1800 MHz		1.4	44 h			x	no	5-10 donors	<0.05	yes	yes	no	no	yes
	Human peripheral blood mononuclear cells	x		900 MHz		0.073	Intermittent			x	no	25 donors	<0.05	yes	yes	no	no	yes
	Human peripheral blood mononuclear cells	x		900 MHz		0.073	Intermittent			x	no	25 donors	<0.05	yes	yes	no	no	yes
	Epidermoid carcinoma KB cells		x	1950 MHz		3.6	1 h	x	yes			3	<0.05	no	no	no	no	yes
[15]	Epidermoid carcinoma KB cells		x	1950 MHz		3.6	2 h	x	yes			3	<0.05	no	no	no	no	yes
	Epidermoid carcinoma KB cells		x	1950 MHz		3.6	3 h	x	yes			3	<0.05	no	no	no	no	yes
	HL-60 (human acute myeloid leukaemia cells)		x	1900 MHz		1	6 h	x	no			5	<0.05	yes	yes	no	no	no
[16]	HL-60 (human acute myeloid leukaemia cells)		x	1900 MHz		10	6 h	x	no			5	<0.05	yes	yes	no	no	no
	TK6 cells (lymphoblastoma cells)		x	1900 MHz		1	6 h	x	no			5	<0.05	yes	yes	no	no	no

	TK6 cells (lymphoblastoma cells)		x	1900 MHz	10	6 h	x	no			5	<0.05	yes	yes	no	no	no
	Mono Mac 6 cells (human monocytes),		x	1900 MHz	1	6 h	x	no			5	<0.05	yes	yes	no	no	no
	Mono Mac 6 cells (human monocytes),		x	1900 MHz	10	6 h	x	no			5	<0.05	yes	yes	no	no	no
	HL-60 (human acute myeloid leukaemia cells)		x	1900 MHz	1	6 h			x	no	5	<0.05	yes	yes	no	no	no
	HL-60 (human acute myeloid leukaemia cells)		x	1900 MHz	10	6 h			x	no	5	<0.05	yes	yes	no	no	no
	TK6 cells (lymphoblastoma cells)		x	1900 MHz	1	6 h			x	no	5	<0.05	yes	yes	no	no	no
	TK6 cells (lymphoblastoma cells)		x	1900 MHz	10	6 h			x	no	5	<0.05	yes	yes	no	no	no
	Mono Mac 6 cells (human monocytes),		x	1900 MHz	1	6 h			x	no	5	<0.05	yes	yes	no	no	no
	Mono Mac 6 cells (human monocytes),		x	1900 MHz	10	6 h			x	no	5	<0.05	yes	yes	no	no	no
[17]	Embryonic mouse neural stem cells (eNSCs)		x	1800 MHz	4	Intermittent	x	no			4	<0.05	yes	yes	yes	yes	yes
	Embryonic mouse neural stem cells (eNSCs)		x	1800 MHz	4	Intermittent			x	no	4	<0.05	yes	yes	no	yes	yes
	Embryonic mouse neural stem cells (eNSCs)		x	1800 MHz	4	Intermittent			x	no	4	<0.05	yes	yes	no	yes	yes
	Embryonic mouse neural stem cells (eNSCs)		x	1800 MHz	2	Intermittent			x	no	4	<0.05	yes	yes	no	yes	yes
	Embryonic mouse neural stem cells (eNSCs)		x	1800 MHz	1	Intermittent			x	no	4	<0.05	yes	yes	no	yes	yes
	Embryonic mouse neural stem cells (eNSCs)		x	1800 MHz	4	Intermittent			x	no	4	<0.05	yes	yes	yes	yes	yes
	Cytolytic T lymphocytes		x	2450 MHz	50	2 h			x	yes	1	<0.05	yes	no	yes	no	yes
[18]	CHO (Chinese hamster ovary) cells		x	27 MHz	5	2 h			x	yes	2; 7	<0.05	yes	no	no	no	yes
	CHO (Chinese hamster ovary) cells		x	2452 MHz	25	2 h			x	yes	2; 7	<0.05	yes	no	no	no	yes
	CHO (Chinese hamster ovary) cells		x	27 MHz	5	2 h			x	yes	2; 7	<0.05	yes	no	no	no	yes
	CHO (Chinese hamster ovary) cells		x	2453 MHz	25	2 h			x	yes	2; 7	<0.05	yes	no	no	no	yes
[19]	ES p53+	x		1710 MHz	1.5	48 h	x	no			2	no	yes	yes	no	yes	yes
	ES p53-	x		1710 MHz	1.5	48 h	x	yes			2	no	yes	yes	no	yes	yes
	ES p53+	x		1710 MHz	0.4	48 h	x	no			2	no	yes	yes	no	yes	yes
	ES p53-	x		1710 MHz	0.4	48 h	x	no			2	no	yes	yes	no	yes	yes
	ES p53+	x		1710 MHz	0.11	6 h	x	no			2	no	yes	yes	no	yes	yes
	ES p53-	x		1710 MHz	0.11	6 h	x	yes			2	no	yes	yes	no	yes	yes
	ES p53+	x		1710 MHz	1.5	48 h			x	no	2	no	yes	yes	no	yes	yes

	ES p53-	x		1710 MHz		1.5	48 h			x	yes	2	no	yes	yes	no	yes	yes
	ES p53+	x		1710 MHz		0.4	48 h			x	no	2	no	yes	yes	no	yes	yes
	ES p53-	x		1710 MHz		0.4	48 h			x	no	2	no	yes	yes	no	yes	yes
	ES p53+	x		1710 MHz		0.11	6 h			x	no	2	no	yes	yes	no	yes	yes
	ES p53-	x		1710 MHz		0.11	6 h			x	yes	2	no	yes	yes	no	yes	yes
[20]	SN56 cholinergic cell line		x	900 MHz		1	24 h			x	no	4	<0.05	yes	yes	no	yes	yes
	SN56 cholinergic cell line		x	900 MHz		1	48 h			x	no	4	<0.05	yes	yes	no	yes	yes
	SN56 cholinergic cell line		x	900 MHz		1	72 h			x	no	4	<0.05	yes	yes	no	yes	yes
	Rat primary cortical neurons	x		900 MHz		1	96 h			x	yes	4	<0.05	yes	yes	no	yes	yes
[21]	Human keratinocytes (HaCaT)		x	900 MHz		0.04	18 h			x	yes	3	no	yes	yes	no	no	yes
	Human keratinocytes (HaCaT)		x	900 MHz		0.08	18 h			x	yes	3	no	yes	yes	no	no	yes
[22]	Human peripheral blood lymphocytes (hPBLs)	x		1800 MHz		0.21	6 h			x	yes	1 (in treeplicates)	<0.05	yes	yes	no	no	yes
	Human peripheral blood lymphocytes (hPBLs)	x		1800 MHz		0.21	8 h			x	yes	1 (in treeplicates)	<0.05	yes	yes	no	no	yes
	Human peripheral blood lymphocytes (hPBLs)	x		1800 MHz		0.21	24 h			x	yes	1 (in treeplicates)	<0.05	yes	yes	no	no	yes
	Human peripheral blood lymphocytes (hPBLs)	x		1800 MHz		0.21	48 h			x	yes	1 (in treeplicates)	<0.05	yes	yes	no	no	yes
[23]	Spermatozoa	x		900 MHz		2	1 h	x	no			12	no	no	yes	no	no	yes
	Spermatozoa	x		900 MHz		5.7	1 h	x	no			12	no	no	yes	no	no	yes
[24]	U-87 MG, human astrocytoma cell line		x	835 MHz	40 W/cm^{-2}		Intermittent			x	no	8	0.018	yes	no	no	no	yes
	U-87 MG, human astrocytoma cell line		x	835 MHz			Intermittent			x	yes	8	0.02	yes	no	no	no	yes
[25]	U937		x	900 MHz		0.2	2 h	x	no			6	<0.05	yes	yes	yes	no	yes
	SK-N-SH (human neuroblastoma) cell line		x	900 MHz		0.2	2 h			x	no	6	<0.05	yes	yes	yes	no	yes
[26]	C3h 10t1⁄2		x	835.62 MHz		0.6	13 h			x	no	3	<0.05	yes	yes	no	no	yes
	C3h 10t1⁄3		x	835.62 MHz		0.6	100 h			x	no	4	<0.05	yes	yes	no	no	yes
	C3h 10t1⁄4		x	847.74 MHz		0.6	13 h			x	no	5	<0.05	yes	yes	no	no	yes
	C3h 10t1⁄5		x	847.74 MHz		0.6	100 h			x	no	6	<0.05	yes	yes	no	no	yes
	U87mg		x	835.62 MHz		0.6	13 h			x	no	7	<0.05	yes	yes	no	no	yes
	U87mg		x	835.62 MHz		0.6	100 h			x	no	8	<0.05	yes	yes	no	no	yes
	U87mg		x	847.74 MHz		0.6	13 h			x	no	9	<0.05	yes	yes	no	no	yes
	U87mg		x	847.74 MHz		0.6	100 h			x	no	3	<0.05	yes	yes	no	no	yes
[27]	Human hacat cells, AL cells (human-hamster hybrid cells)		x	900 MHz	5 V/m		30 min			x	no	3	<0.05	no	no	no	yes	yes
	Human hacat cells, AL cells (human-hamster hybrid cells)		x	900 MHz	10 V/m		30 min			x	no	3	<0.05	no	no	no	yes	yes

	Human hacat cells, AL cells (human-hamster hybrid cells)	x	900 MHz	30 V/m		30 min			x	no	3	<0.05	no	no	no	yes	yes
	Human hacat cells, AL cells (human-hamster hybrid cells)	x	900 MHz	90 V/m		30 min			x	no	3	<0.05	no	no	no	yes	yes
[28]	Hacat human dermal fibroblasts	x	106 GHz	0.04 mW/cm²		2 h			x	no	3	no	yes	no	yes	yes	yes
	Hacat human dermal fibroblasts	x	106 GHz	0.39 mW/cm²		2 h			x	no	3	no	yes	no	yes	yes	yes
	Hacat human dermal fibroblasts	x	106 GHz	0.88 mW/cm²		2 h			x	no	3	no	yes	no	yes	yes	yes
	Hacat human dermal fibroblasts	x	106 GHz	0.88 mW/cm²		8 h			x	no	3	no	yes	no	yes	yes	yes
[29]	A172 glioblastoma	x	2140 MHz		0.08	24 h	x	no			2 with triplicates	<0.01	yes	yes	no	yes	yes
	A172 glioblastoma	x	2140 MHz		0.08	48 h	x	no			2 with triplicates	<0.01	yes	yes	no	yes	yes
	A172 glioblastom	x	2140 MHz		0.25	24 h	x	no			2 with triplicates	<0.01	yes	yes	no	yes	yes
	A172 glioblastom	x	2140 MHz		0.8	24 h	x	no			2 with triplicates	<0.01	yes	yes	no	yes	yes
	A172 glioblastom	x	2140 MHz		0.08	24 h	x	no			2 with triplicates	<0.01	yes	yes	no	yes	yes
	IMR-90 fibroblasts	x	2140 MHz		0.08	28 h	x	no			2 with triplicates	<0.01	yes	yes	no	yes	yes
	IMR-90 fibroblasts	x	2140 MHz		0.08	28 h	x	no			2 with triplicates	<0.01	yes	yes	no	yes	yes
	A172 glioblastoma	x	2140 MHz		0.08	24 h			x	no	2 with triplicates	<0.01	yes	yes	no	yes	yes
	A172 glioblastoma	x	2140 MHz		0.08	48 h			x	no	2 with triplicates	<0.01	yes	yes	no	yes	yes
	A172 glioblastoma	x	2140 MHz		0.25	24 h			x	no	2 with triplicates	<0.01	yes	yes	no	yes	yes
	A172 glioblastoma	x	2140 MHz		0.8	24 h			x	no	2 with triplicates	<0.01	yes	yes	no	yes	yes
	A172 glioblastoma	x	2140 MHz		0.08	24 h			x	no	2 with triplicates	<0.01	yes	yes	no	yes	yes
	IMR-90 fibroblasts	x	2140 MHz		0.08	28 h			x	no	2 with triplicates	<0.01	yes	yes	no	yes	yes
	IMR-90 fibroblasts	x	2140 MHz		0.08	28 h			x	no	2 with triplicates	<0.01	yes	yes	no	yes	yes
[30]	Murine L929 fibroblast cells	x	872 MHz		5	1 h	x	no			6	<0.05	yes	yes	yes	yes	yes
	Murine L929 fibroblast cells	x	872 MHz		5	24 h	x	no			3	<0.05	yes	yes	yes	yes	yes
	Murine L929 fibroblast cells	x	872 MHz		5	1 h	x	no			6	<0.05	yes	yes	yes	yes	yes
	Murine L929 fibroblast cells	x	872 MHz		5	24 h	x	no			4	<0.05	yes	yes	yes	yes	yes
[31]	SH-SY5Y cell line (human neuroblastoma)	x	872 MHz		5	2 h			x	no	3	<0.05	yes	yes	yes	no	yes
	SH-SY5Y cell line (human neuroblastoma)	x	872 MHz		5	24 h			x	no	3	<0.05	yes	yes	yes	no	yes

	SH-SY5Y cell line (human neuroblastoma)		x	872 MHz	5	2 h			x	no	3	<0.05	yes	yes	yes	no	yes
	SH-SY5Y cell line (human neuroblastoma)		x	872 MHz	5	24 h			x	no	3	<0.05	yes	yes	yes	no	yes
	L929 (mouse fibroblasts)		x	872 MHz	5	2 h	x	no			3; 5	<0.05	yes	yes	yes	no	yes
	L929 (mouse fibroblasts)		x	872 MHz	5	24 h	x	no			3; 5	<0.05	yes	yes	yes	no	yes
	L929 (mouse fibroblasts)		x	872 MHz	5	2 h	x	yes			3; 5	<0.05	yes	yes	yes	no	yes
	L929 (mouse fibroblasts)		x	872 MHz	5	24 h	x	yes			3; 5	<0.05	yes	yes	yes	no	yes
[30]	Murine L929 fibroblast cells		x	872 MHz	5	1 h			x	no	6	<0.05	yes	yes	yes	yes	yes
	Murine L929 fibroblast cells		x	872 MHz	5	24 h			x	no	3	<0.05	yes	yes	yes	yes	yes
	Murine L929 fibroblast cells		x	872 MHz	5	1 h			x	no	6	<0.05	yes	yes	yes	yes	yes
	Murine L929 fibroblast cells		x	872 MHz	5	24 h			x	no	4	<0.05	yes	yes	yes	yes	yes
[31]	L929 (mouse fibroblasts)		x	872 MHz	5	2 h			x	no	3; 5	<0.05	yes	yes	yes	no	yes
	L929 (mouse fibroblasts)		x	872 MHz	5	24 h			x	no	3; 5	<0.05	yes	yes	yes	no	yes
	L929 (mouse fibroblasts)		x	872 MHz	5	2 h			x	no	3; 5	<0.05	yes	yes	yes	no	yes
	L929 (mouse fibroblasts)		x	872 MHz	5	24 h			x	no	3; 5	<0.05	yes	yes	yes	no	yes
[32]	Jurkat T-cells		x	1763 MHz	2	24 h			x	no	5	<0.05	yes	yes	no	no	yes
	Jurkat T-cells		x	1763 MHz	10	24 h			x	no	5	<0.05	yes	yes	no	no	yes
[33]	Mouse HEI-OC1 auditory hair cells		x	1763 MHz	20	24 h			x	no	3	<0.05	yes	yes	no	no	yes
	Mouse HEI-OC1 auditory hair cells		x	1763 MHz	20	48 h			x	no	3	<0.05	yes	yes	no	no	yes
[34]	HL-60 cells		x	900 MHz	0.0012	Intermittent	x	yes			3	<0.05	yes	no	yes	no	yes
	HL-60 cells		x	900 MHz	0.0012	Intermittent			x	yes	3	<0.05	yes	no	yes	no	yes
[35]	Human neuroblastoma cells SH-SY5Y		x	900 MHz	0.25	24 h	x	no			3	<0.05	yes	yes	yes	yes	yes
	Human neuroblastoma cells SH-SY5Y		x	900 MHz	2	24 h	x	no			3	<0.05	yes	yes	yes	yes	yes
	Human neuroblastoma cells SH-SY5Y		x	900 MHz	0.25	24 h	x	no			3	<0.05	yes	yes	yes	yes	yes
	Human neuroblastoma cells SH-SY5Y		x	900 MHz	2	24 h	x	no			3	<0.05	yes	yes	yes	yes	yes
[36]	Cortices of embryonic Wistar rats	x		900 MHz	0.25	24 h	x	no			3	<0.05	yes	yes	yes	yes	yes
[37]	Rat primary neuronal cultures	x		900 MHz	2	24 h	x	yes			3	<0.05	yes	yes	yes	yes	yes

[38]	Breast cancer cells (mda-mb-231)		x	900 MHz	0.36	1 h	x	yes			3; 8	<0.05	no	yes	no	no	yes
[39]	Mouse brain	x		10.715 GHz	0.725	Intermittent	x	yes			1	<0.05	no	yes	no	yes	yes
[40]	Rat bone marrow cells	x		900 MHz	2	30 min			x	no	14	<0.05	yes	yes	yes	yes	yes
	Rat lymphocytes	x		900 MHz	2	30 min			x	no	11	<0.05	yes	yes	yes	yes	yes
[41]	AMA cells (transformed human epithelial amnion cells)		x	837 MHz	4	1 h			x	no	3	<0.05	yes	yes	no	no	yes
	AMA cells (transformed human epithelial amnion cells)		x	837 MHz combined with 1950 MHz	4	1 h			x	no	3	<0.05	yes	yes	no	no	yes
[42]	Mono Mac 6 cells (human monocytes)		x	1800 MHz	2	12 h	x	no			3	<0.05	yes	yes	yes	yes	yes
	Mono Mac 6 cells (human monocytes)		x	1800 MHz	2	12 h			x	no	3	<0.05	yes	yes	yes	yes	yes
[43]	NIH3T3 cells (mouse fibroblasts)		x	849 MHz	2	1 h			x	no	4	<0.05; <0.01	yes	yes	yes	no	yes
	NIH3T3 cells (mouse fibroblasts)		x	850 MHz	10	1 h			x	no	4	<0.05; <0.01	yes	yes	yes	no	yes
	NIH3T3 cells (mouse fibroblasts)		x	850 MHz	2	Intermittent			x	no	4	<0.05; <0.01	yes	yes	yes	no	yes
	NIH3T3 cells (mouse fibroblasts)		x	850 MHz	10	Intermittent			x	no	4	<0.05; <0.01	yes	yes	yes	no	yes
[44]	MCF-7 (human breast cancer cell line)		x	837 MHz	4	1 h			x	no	3	<0.05	yes	yes	yes	yes	yes
	MCF-7 (human breast cancer cell line)		x	837 MHz combined with 1950 MHz	4	1 h			x	no	3	<0.05	yes	yes	yes	yes	yes
[45]	HL-60 cell		x	2450 MHz	10	2 h	x	yes			n.s.	<0.05	yes	no	no	no	yes
	HL-60 cell		x	2450 MHz	10	6 h	x	yes			n.s.	<0.05	yes	no	no	no	yes
	HL-60 cell		x	2450 MHz	10	2 h			x	yes	n.s.	<0.05	yes	no	no	no	yes
	HL-60 cell		x	2450 MHz	10	6 h			x	yes	n.s.	<0.05	yes	no	no	no	yes
[46]	Adipose-derived stem cells (ASC)	x		2450 MHz	0.24	Intermittent	x	no			5	<0.05	no	no	no	no	yes
	Adipose-derived stem cells (ASC)	x		2450 MHz	0.24	Intermittent			x	no	5	<0.05	no	no	no	no	yes
[47]	Mouse spermatocytes (GC2)		x	1800 MHz	14	Intermittent	x	no			3	<0.05	yes	yes	yes	yes	yes
[48]	Rat primary,cortical neurons	x		1800 MHz		24 h	x	yes			3	<0.05	no	no	no	no	No
[49]	Rat astrocytes	x		1950 MHz	5.36	12 h	x	no			3	<0.05	no	no	no	no	yes
	Rat astrocytes	x		1950 MHz	5.36	24 h	x	no			3	<0.05	no	no	no	no	yes
	Rat astrocytes	x		1950 MHz	5.36	48 h	x	yes			3	<0.05	no	no	no	no	yes
	C6 cells (rat glioma cell line)		x	1950 MHz	5.36	12 h	x	no			3	<0.05	no	no	no	no	yes
	C6 cells (rat glioma cell line)		x	1950 MHz	5.36	24 h	x	no			3	<0.05	no	no	no	no	yes
	C6 cells (rat glioma cell line)		x	1950 MHz	5.36	48 h	x	no			3	<0.05	no	no	no	no	yes
	Rat primary,cortical neurons	x		1800 MHz		24 h			x	yes	3	<0.05	no	no	no	no	No
	Rat astrocytes	x		1950 MHz	5.36	12 h			x	no	3	<0.05	no	no	no	no	yes

	Rat astrocytes	x		1950 MHz	5.36	24 h			x	no	3	<0.05	no	no	no	no	yes
	Rat astrocytes	x		1950 MHz	5.36	48 h			x	yes	3	<0.05	no	no	no	no	yes
	C6 cells (rat glioma cell line)		x	1950 MHz	5.36	12 h			x	no	3	<0.05	no	no	no	no	yes
	C6 cells (rat glioma cell line)		x	1950 MHz	5.36	24 h			x	no	3	<0.05	no	no	no	no	yes
	C6 cells (rat glioma cell line)		x	1950 MHz	5.36	48 h			x	no	3	<0.05	no	no	no	no	yes
[50]	SRA01/04-HLEC (human eye lens epithelial cells)	x		1800 MHz	1	2 h			x	no	100 cells	<0.05	yes	yes	yes	no	yes
	SRA01/04-HLEC (human eye lens epithelial cells)	x		1800 MHz	2	2 h			x	no	100 cells	<0.05	yes	yes	yes	no	yes
	SRA01/04-HLEC (human eye lens epithelial cells)	x		1800 MHz	3	3 h			x	no	100 cells	<0.05	yes	yes	yes	no	yes
[51]	Human peripheral blood mononuclear cells (PBMC)	x		900 MHz	0.43	1 h	x	no			5	<0.05	no	no	no	no	no
	Human peripheral blood mononuclear cells (PBMC)	x		900 MHz	0.43	2 h	x	yes			3	<0.05	no	no	no	no	no
	Human peripheral blood mononuclear cells (PBMC)	x		900 MHz	0.43	4 h	x	yes			3	<0.05	no	no	no	no	no
	Human peripheral blood mononuclear cells (PBMC)	x		900 MHz	0.43	6 h	x	yes			3	<0.05	no	no	no	no	no
	Human peripheral blood mononuclear cells (PBMC)	x		900 MHz	0.43	8 h	x	yes			3	<0.05	no	no	no	no	no
[52]	Human peripheral blood lymphocytes	x		954 MHz	1.5	2 h			x	no	8	<0.05	no	no	yes	no	yes
[53]	Human skin fibroblasts		x	2400 MHz	0.00028	72 h	x	no			6	<0.05	no	no	no	no	no
	Human skin fibroblasts		x	2400 MHz	0.00028	72 h			x	no	6	<0.05	no	no	no	no	no
[54]	T-lymphoblastoid leukemia CCRF-CEM		x	900 MHz	0.0035	2 h	x	yes			1	<0.05	yes	yes	no	no	yes
	T-lymphoblastoid leukemia CCRF-CEM		x	900 MHz	0.0035	4 h	x	yes			1	<0.05	yes	yes	no	no	yes
	T-lymphoblastoid leukemia CCRF-CEM		x	900 MHz	0.0035	12 h	x	yes			1	<0.05	yes	yes	no	no	yes
	T-lymphoblastoid leukemia CCRF-CEM		x	900 MHz	0.0035	24 h	x	no			1	<0.05	yes	yes	no	no	yes
	T-lymphoblastoid leukemia CCRF-CEM		x	900 MHz	0.0035	48 h	x	no			1	<0.05	yes	yes	no	no	yes
	T-lymphoblastoid leukemia CCRF-CEM		x	900 MHz	0.0035	2 h			x	no	1	<0.05	yes	yes	no	no	yes
	T-lymphoblastoid leukemia CCRF-CEM		x	900 MHz	0.0035	4 h			x	no	1	<0.05	yes	yes	no	no	yes
	T-lymphoblastoid leukemia CCRF-CEM		x	900 MHz	0.0035	12 h			x	no	1	<0.05	yes	yes	no	no	yes
	T-lymphoblastoid leukemia CCRF-CEM		x	900 MHz	0.0035	24 h			x	no	1	<0.05	yes	yes	no	no	yes
	T-lymphoblastoid leukemia CCRF-CEM		x	900 MHz	0.0035	48 h			x	no	1	<0.05	yes	yes	no	no	yes

[55]	Human neuroblastoma cell line LAN-5		x	900 MHz	1	24 h	x	no			3	<0.05	yes	yes	yes	yes	yes
	Human neuroblastoma cell line LAN-5		x	900 MHz	1	48 h	x	no			3	<0.05	yes	yes	yes	yes	yes
	Human neuroblastoma cell line LAN-5		x	900 MHz	1	72 h	x	no			3	<0.05	yes	yes	yes	yes	yes
	Human neuroblastoma cell line LAN-5		x	900 MHz	1	24 h			x	no	3	<0.05	yes	yes	yes	yes	yes
	Human neuroblastoma cell line LAN-5		x	900 MHz	1	48 h			x	no	3	<0.05	yes	yes	yes	yes	yes
	Human neuroblastoma cell line LAN-5		x	900 MHz	1	72 h			x	no	3	<0.05	yes	yes	yes	yes	yes
[56]	MO54 (human glioma cells)		x	1950 MHz	2	2 h			x	no	1	<0.05	yes	yes	yes	no	yes
	MO54 (human glioma cells)		x	1950 MHz	10	2 h			x	no	2	<0.05	yes	yes	yes	no	yes
[57]	B16F10 (murine melanoma cells)		x	900 MHz	2.2	1 h			x	no	n.s.	<0.05	no	yes	no	no	yes
[58]	(N2a) murine neuroblastoma cell line		x	935 MHz	2	24 h	x	no			3	<0.05	yes	yes	no	no	yes
	(N2a) murine neuroblastoma cell line		x	935 MHz	2	24 h	x	no			3	<0.05	yes	yes	no	no	yes
	(N2a) murine neuroblastoma cell line		x	935 MHz	2	24 h	x	no			3	<0.05	yes	yes	no	no	yes
[59]	HL-60 cell line		x	2450 MHz	0.1	1 h			x	yes	6	<0.001	no	no	no	no	no
	HL-60 cell line		x	2450 MHz	0.1	2 h			x	yes	6	<0.001	no	no	no	no	no
	HL-60 cell line		x	2450 MHz	0.1	12 h			x	yes	6	<0.001	no	no	no	no	no
	HL-60 cell line		x	2450 MHz	0.1	24 h			x	yes	6	<0.001	no	no	no	no	no
[60]	ES R1 cells (mouse embryonic stem cells)	x		1710 MHz	1.5	6 h	x	yes			5	no	yes	yes	no	yes	yes
	ES R1 cells (mouse embryonic stem cells)	x		1710 MHz	1.5	24 h	x	yes			5	no	yes	yes	no	yes	yes
	ES-derived nestin-positive neural progenitor cells	x		1710 MHz	1.5	6 h	x	yes			5	no	yes	yes	no	yes	yes
	ES-derived nestin-positive neural progenitor cells	x		1710 MHz	1.5	24 h	x	yes			5	no	yes	yes	no	yes	yes
	ES R1 cells (mouse embryonic stem cells)	x		1710 MHz	1.5	6 h			x	no	5	no	yes	yes	no	yes	yes
	ES R1 cells (mouse embryonic stem cells)	x		1710 MHz	1.5	24 h			x	no	5	no	yes	yes	no	yes	yes

	ES-derived nestin-positive neural progenitor cells	x		1710 MHz	1.5	6 h			x	no	5	no	yes	yes	no	yes	yes
	ES-derived nestin-positive neural progenitor cells	x		1710 MHz	1.5	24 h			x	no	5	no	yes	yes	no	yes	yes
[61]	Hepatocarcinoma (hepg2)		x	900 MHz	2	Intermittent	x	no			4	<0.05	yes	yes	no	yes	yes
	Hepatocarcinoma (HepG2)		x	900 MHz	2	Intermittent	x	no			4	<0.05	yes	yes	no	yes	yes
	Hepatocarcinoma (HepG2)		x	900 MHz	2	Intermittent	x	no			4	<0.05	yes	yes	no	yes	yes
	Hepatocarcinoma (HepG2)		x	900 MHz	2	Intermittent	x	yes			4	<0.05	yes	yes	no	yes	yes
	Hepatocarcinoma (HepG2)		x	1800 MHz	2	Intermittent	x	no			4	<0.05	yes	yes	no	yes	yes
	Hepatocarcinoma (HepG2)		x	1800 MHz	2	Intermittent	x	no			4	<0.05	yes	yes	no	yes	yes
	Hepatocarcinoma (HepG2)		x	1800 MHz	2	Intermittent	x	no			4	<0.05	yes	yes	no	yes	yes
	Hepatocarcinoma (HepG2)		x	1800 MHz	2	Intermittent	x	yes			4	<0.05	yes	yes	no	yes	yes
	Hepatocarcinoma (HepG2)		x	900 MHz	2	Intermittent			x	no	4	<0.05	yes	yes	no	yes	yes
	Hepatocarcinoma (HepG2)		x	900 MHz	2	Intermittent			x	no	4	<0.05	yes	yes	no	yes	yes
	Hepatocarcinoma (HepG2)		x	900 MHz	2	Intermittent			x	no	4	<0.05	yes	yes	no	yes	yes
	Hepatocarcinoma (HepG2)		x	900 MHz	2	Intermittent			x	yes	4	<0.05	yes	yes	no	yes	yes
	Hepatocarcinoma (HepG2)		x	1800 MHz	2	Intermittent			x	yes	4	<0.05	yes	yes	no	yes	yes
	Hepatocarcinoma (HepG2)		x	1800 MHz	2	Intermittent			x	no	4	<0.05	yes	yes	no	yes	yes
	Hepatocarcinoma (HepG2)		x	1800 MHz	2	Intermittent			x	no	4	<0.05	yes	yes	no	yes	yes
	Hepatocarcinoma (HepG2)		x	1800 MHz	2	Intermittent			x	yes	4	<0.05	yes	yes	no	yes	yes
[62]	Detroit 550 (normal human skin fibroblasts)	x		902.4 MHz	0.6	1 h			x	yes	6	no	yes	no	no	no	no
[63]	Jurkat cells (human lymphoblastoid T cells)		x	900 MHz	1.35	1 h	x	no			3	<0.05	yes	yes	yes	yes	yes
	Pbls (human peripheral blood lymphocytes)	x		900 MHz	1.35	1 h	x	no			3	<0.05	yes	yes	yes	yes	yes
	Jurkat cells (human lymphoblastoid T cells)		x	900 MHz	1.35	1 h			x	yes	3	<0.05	yes	yes	yes	yes	yes
	Pbls (human peripheral blood lymphocytes)	x		900 MHz	1.35	1 h			x	no	3	<0.05	yes	yes	yes	yes	yes
[64]	V79 (Chinese hamster fibroblast cells)		x	864 MHz	0.08	1 h			x	no	n.s.	<0.05	yes	no	no	no	yes
	V79 (Chinese hamster fibroblast cells)		x	864 MHz	0.08	2 h			x	yes	n.s.	<0.05	yes	no	no	no	yes

	V79 (Chinese hamster fibroblast cells)		x	864 MHz		0.08	3 h			x	yes	n.s.	<0.05	yes	no	no	no	yes
[65]	V79 (Chinese hamster fibroblast cells)		x	935 MHz		0.12	1 h			x	no	3	<0.05	no	no	yes	no	yes
	V79 (Chinese hamster fibroblast cells)		x	935 MHz		0.12	2 h			x	no	3	<0.05	no	no	yes	no	yes
	V79 (Chinese hamster fibroblast cells)		x	935 MHz		0.12	3 h			x	yes	3	<0.05	no	no	yes	no	yes
[66]	V79 (Chinese hamster fibroblast cells)		x	864 MHz		0.08	1 h			x	no	3	<0.05	no	no	yes	no	yes
	V79 (Chinese hamster fibroblast cells)		x	864 MHz		0.08	2 h			x	yes	3	<0.05	no	no	yes	no	yes
	V79 (Chinese hamster fibroblast cells)		x	864 MHz		0.08	3 h			x	yes	3	<0.05	no	no	yes	no	yes
	V79 (Chinese hamster fibroblast cells)		x	935 MHz		0.12	1 h			x	no	3	<0.05	no	no	yes	no	yes
	V79 (Chinese hamster fibroblast cells)		x	935 MHz		0.12	2 h			x	no	3	<0.05	no	no	yes	no	yes
	V79 (Chinese hamster fibroblast cells)		x	935 MHz		0.12	3 h			x	yes	3	<0.05	no	no	yes	no	yes
[67]	Jurkat cells (clone E6-1)		x	2450 MHz	5 mW/cm^2		48 h	x	yes			3	<0.001	yes	no	no	no	yes
	Jurkat cells (clone E6-1)		x	2450 MHz	5 mW/cm^2		48 h			x	no	3	<0.001	yes	no	no	no	yes
[68]	Human astrocytoma cell		x	9600 MHz		0.0004	15 min	x	no			2	<0.05	yes	yes	no	yes	yes
	Human astrocytoma cell		x	9600 MHz		0.0004	30 min	x	no			2	<0.05	yes	yes	no	yes	yes
	Human astrocytoma cell		x	9600 MHz		0.0004	1 h	x	no			2	<0.05	yes	yes	no	yes	yes
	Human astrocytoma cell		x	9600 MHz		0.0004	24 h	x	no			2	<0.05	yes	yes	no	yes	yes
	Human astrocytoma cell		x	9600 MHz		0.0004	15 min			x	no	2	<0.05	yes	yes	no	yes	yes
	Human astrocytoma cell		x	9600 MHz		0.0004	30 min			x	no	2	<0.05	yes	yes	no	yes	yes
	Human astrocytoma cell		x	9600 MHz		0.0004	1 h			x	no	2	<0.05	yes	yes	no	yes	yes
	Human astrocytoma cell		x	9600 MHz		0.0004	24 h			x	yes	2	<0.05	yes	yes	no	yes	yes
[69]	Human leukemia cells (HL60)		x	400 MHz			24 h	x	no			3	<0.05	no	no	no	no	no
	Human leukemia cells (HL60)		x	400 MHz			72 h	x	no			3	<0.05	no	no	no	no	no
[70]	Isolated human skin cells	x		900 MHz		2	48 h	x	no			5	<0.05	yes	yes	yes	no	yes

	Human reconstructed epidermis (HRE) using human keratinocytes	x		900 MHz	2	48 h	x	no			5	<0.05	yes	yes	yes	no	yes
	Isolated human skin cells	x		900 MHz	2	48 h			x	no	5	<0.05	yes	yes	yes	no	yes
	Human reconstructed epidermis (HRE) using human keratinocytes	x		900 MHz	2	48 h			x	no	5	<0.05	yes	yes	yes	no	yes
[71]	Human blood lymphocytes	x		1950 MHz	0.3	20 h			x	no	4	<0.05	yes	yes	yes	yes	yes
[72]	Human blood lymphocytes	x		900 MHz	1.25	20 h			x	no	3	<0.05	yes	yes	yes	yes	yes
	Human blood lymphocytes	x		900 MHz	1.25	20 h			x	no	3	<0.05	yes	yes	yes	yes	yes
	Human blood lymphocytes	x		900 MHz	1.25	20 h			x	no	3	<0.05	yes	yes	yes	yes	yes
[73]	Human blood lymphocytes	x		900 MHz	1.25	20 h			x	no	5	<0.05	yes	yes	yes	yes	yes
[74]	Human healthy dermal fibroblasts		x	900 MHz	1	24 h			x	no	3	<0.05	yes	yes	yes	yes	yes
	Human dermal fibroblasts from Turner syndrome patients		x	900 MHz	1	24 h			x	no	3	<0.05	yes	yes	yes	yes	yes
[75]	Human peripheral blood lymphocytes	x		900 MHz	1	24 h			x	no	5	<0.05	yes	yes	yes	yes	yes
	Human peripheral blood lymphocytes	x		900 MHz	5	24 h			x	no	5	<0.05	yes	yes	yes	yes	yes
	Human peripheral blood lymphocytes	x		900 MHz	10	24 h			x	no	5	<0.05	yes	yes	yes	yes	yes
[76]	Human-hamster hybrid (A(L)) cell line		x	900 MHz	11.5	30 min			x	no	3	<0.05	yes	yes	yes	yes	yes
[77]	FC2 cell hybrid		x	900 MHz	0.06	30 min			x	yes	2	<0.05	yes	yes	no	no	yes
	FC2 cell hybrid		x	900 MHz	0.06	2 h			x	yes	2	<0.05	yes	yes	no	no	yes
[78]	Human A172 (glioblastoma)		x	2142.5 MHz	0.08	24 h			x	no	2 to 4	<0.05	yes	yes	yes	yes	yes
	Human A172 (glioblastoma)		x	2142.5 MHz	0.08	48 h			x	no	2 to 4	<0.05	yes	yes	yes	yes	yes
	Human A172 (glioblastoma)		x	2142.5 MHz	0.08	72 h			x	no	2 to 4	<0.05	yes	yes	yes	yes	yes
	Human A172 (glioblastoma)		x	2142.5 MHz	0.08	96 h			x	no	2 to 4	<0.05	yes	yes	yes	yes	yes
	Human A172 (glioblastoma)		x	2142.5 MHz	0.25	24 h			x	no	2 to 4	<0.05	yes	yes	yes	yes	yes
	Human A172 (glioblastoma)		x	2142.5 MHz	0.25	48 h			x	no	2 to 4	<0.05	yes	yes	yes	yes	yes
	Human A172 (glioblastoma)		x	2142.5 MHz	0.25	72 h			x	no	2 to 4	<0.05	yes	yes	yes	yes	yes
	Human A172 (glioblastoma)		x	2142.5 MHz	0.25	96 h			x	no	2 to 4	<0.05	yes	yes	yes	yes	yes

Human A172 (glioblastoma)	x	2142.5 MHz	0.8	24 h	x	no	2 to 4	<0.05	yes	yes	yes	yes	yes
Human A172 (glioblastoma)	x	2142.5 MHz	0.8	48 h	x	no	2 to 4	<0.05	yes	yes	yes	yes	yes
Human A172 (glioblastoma)	x	2142.5 MHz	0.8	72 h	x	no	2 to 4	<0.05	yes	yes	yes	yes	yes
Human A172 (glioblastoma)	x	2142.5 MHz	0.8	96 h	x	no	2 to 4	<0.05	yes	yes	yes	yes	yes
Human H4 (neuroglioma)	x	2142.5 MHz	0.08	24 h	x	no	2 to 4	<0.05	yes	yes	yes	yes	yes
Human H4 (neuroglioma)	x	2142.5 MHz	0.08	48 h	x	no	2 to 4	<0.05	yes	yes	yes	yes	yes
Human H4 (neuroglioma)	x	2142.5 MHz	0.08	72 h	x	no	2 to 4	<0.05	yes	yes	yes	yes	yes
Human H4 (neuroglioma)	x	2142.5 MHz	0.08	96 h	x	no	2 to 4	<0.05	yes	yes	yes	yes	yes
Human H4 (neuroglioma)	x	2142.5 MHz	0.25	24 h	x	no	2 to 4	<0.05	yes	yes	yes	yes	yes
Human H4 (neuroglioma)	x	2142.5 MHz	0.25	48 h	x	no	2 to 4	<0.05	yes	yes	yes	yes	yes
Human H4 (neuroglioma)	x	2142.5 MHz	0.25	72 h	x	no	2 to 4	<0.05	yes	yes	yes	yes	yes
Human H4 (neuroglioma)	x	2142.5 MHz	0.25	96 h	x	no	2 to 4	<0.05	yes	yes	yes	yes	yes
Human H4 (neuroglioma)	x	2142.5 MHz	0.8	24 h	x	no	2 to 4	<0.05	yes	yes	yes	yes	yes
Human H4 (neuroglioma)	x	2142.5 MHz	0.8	48 h	x	no	2 to 4	<0.05	yes	yes	yes	yes	yes
Human H4 (neuroglioma)	x	2142.5 MHz	0.8	72 h	x	no	2 to 4	<0.05	yes	yes	yes	yes	yes
Human H4 (neuroglioma)	x	2142.5 MHz	0.8	96 h	x	no	2 to 4	<0.05	yes	yes	yes	yes	yes
IMR-90 (fibroblasts from normal fetal lung)	x	2142.5 MHz	0.08	24 h	x	no	2 to 4	<0.05	yes	yes	yes	yes	yes
IMR-90 (fibroblasts from normal fetal lung)	x	2142.5 MHz	0.08	48 h	x	no	2 to 4	<0.05	yes	yes	yes	yes	yes
IMR-90 (fibroblasts from normal fetal lung)	x	2142.5 MHz	0.08	72 h	x	no	2 to 4	<0.05	yes	yes	yes	yes	yes
IMR-90 (fibroblasts from normal fetal lung)	x	2142.5 MHz	0.08	96 h	x	no	2 to 4	<0.05	yes	yes	yes	yes	yes
IMR-90 (fibroblasts from normal fetal lung)	x	2142.5 MHz	0.25	24 h	x	no	2 to 4	<0.05	yes	yes	yes	yes	yes
IMR-90 (fibroblasts from normal fetal lung)	x	2142.5 MHz	0.25	48 h	x	no	2 to 4	<0.05	yes	yes	yes	yes	yes
IMR-90 (fibroblasts from normal fetal lung)	x	2142.5 MHz	0.25	72 h	x	no	2 to 4	<0.05	yes	yes	yes	yes	yes
IMR-90 (fibroblasts from normal fetal lung)	x	2142.5 MHz	0.25	96 h	x	no	2 to 4	<0.05	yes	yes	yes	yes	yes

	IMR-90 (fibroblasts from normal fetal lung)		x	2142.5 MHz	0.8	24 h	x	no	2 to 4	<0.05	yes	yes	yes	yes	yes
	IMR-90 (fibroblasts from normal fetal lung)		x	2142.5 MHz	0.8	48 h	x	no	2 to 4	<0.05	yes	yes	yes	yes	yes
	IMR-90 (fibroblasts from normal fetal lung)		x	2142.5 MHz	0.8	72 h	x	no	2 to 4	<0.05	yes	yes	yes	yes	yes
	IMR-90 (fibroblasts from normal fetal lung)		x	2142.5 MHz	0.8	96 h	x	no	2 to 4	<0.05	yes	yes	yes	yes	yes
[79]	Rat glioma cells (C6)		x	836.55 MHz	0.00059	4 h	x	no	6	<0.05	yes	yes	no	no	yes
	Rat glioma cells (C6)		x	836.55 MHz	0.00059	24 h	x	no	6	<0.05	yes	yes	no	no	yes
	Rat glioma cells (C6)		x	836.55 MHz	0.0059	4 h	x	no	6	<0.05	yes	yes	no	no	yes
	Rat glioma cells (C6)		x	836.55 MHz	0.0059	24 h	x	no	6	<0.05	yes	yes	no	no	yes
	Rat glioma cells (C6)		x	836.55 MHz	0.059	4 h	x	no	6	<0.05	yes	yes	no	no	yes
	Rat glioma cells (C6)		x	836.55 MHz	0.059	24 h	x	no	6	<0.05	yes	yes	no	no	yes
	Primary fetal rat brain culture	x		836.55 MHz	0.00059	4 h	x	no	6	<0.05	yes	yes	no	no	yes
	Primary fetal rat brain culture	x		836.55 MHz	0.00059	24 h	x	no	6	<0.05	yes	yes	no	no	yes
	Primary fetal rat brain culture	x		836.55 MHz	0.0059	4 h	x	no	6	<0.05	yes	yes	no	no	yes
	Primary fetal rat brain culture	x		836.55 MHz	0.0059	24 h	x	no	6	<0.05	yes	yes	no	no	yes
	Primary fetal rat brain culture	x		836.55 MHz	0.059	4 h	x	no	6	<0.05	yes	yes	no	no	yes
	Primary fetal rat brain culture	x		836.55 MHz	0.059	24 h	x	no	6	<0.05	yes	yes	no	no	yes
[80]	Human keratinocyte hacat cells		x	61.2 GHz	770	30 min	x	no	6	<0.05	yes	no	no	yes	no
[81]	Chinese hamster ovary cell line (CHO-K1)		x	2450 MHz	0.05	2 h	x	no	3	no	yes	yes	no	no	yes
	Chinese hamster ovary cell line (CHO-K1)		x	2450 MHz	5	2 h	x	no	3	no	yes	yes	no	no	yes
	Chinese hamster ovary cell line (CHO-K1)		x	2450 MHz	50	2 h	x	no	3	no	yes	yes	no	no	yes
	Chinese hamster ovary cell line (CHO-K1)		x	2450 MHz	100	2 h	x	no	3	no	yes	yes	no	no	yes
	Chinese hamster ovary cell line (CHO-K1)		x	2450 MHz	200	2 h	x	yes	3	no	yes	yes	no	no	yes
	Human malignant glioma (MO54 cells)		x	2450 MHz	0.05	2 h	x	no	3	no	yes	yes	no	no	yes
	Human malignant glioma (MO54 cells)		x	2450 MHz	5	2 h	x	no	3	no	yes	yes	no	no	yes
	Human malignant glioma (MO54 cells)		x	2450 MHz	50	2 h	x	no	3	no	yes	yes	no	no	yes
	Human malignant glioma (MO54 cells)		x	2450 MHz	100	2 h	x	no	3	no	yes	yes	no	no	yes
	Human malignant glioma (MO54 cells)		x	2450 MHz	200	2 h	x	yes	3	no	yes	yes	no	no	yes

[82]	Primary cerebral cortical cells (neurons and astrocytes) of rat embryos	x		900 MHz	0.25	24 h	x	no			3	<0.05	yes	yes	yes	no	yes
	Primary cerebral cortical cells (neurons and astrocytes) of rat embryos	x		900 MHz	0.25	24 h			x	no	3	<0.05	yes	yes	yes	no	yes
[83]	Human neuroblastoma NB69 line		x	2200 MHz	0.023	24 h			x	yes	8	<0.001	yes	yes	no	yes	yes
	Human hepatocarcinoma cell line HEPG2		x	2200 MHz	0.023	24 h			x	no	6	<0.001	yes	yes	no	yes	yes
[84]	Transformed human epithelial amnion cells AMA.		x	960 MHz	0.0021	30 min			x	yes	11	<0.01	yes	yes	no	no	yes
[85]	Human blood lymphocytes	x		2450 MHz	12.46	90 min			x	no	2	<0.05	yes	yes	yes	yes	yes
	Human blood lymphocytes	x		2450 MHz	12.46	Intermittent			x	no	2	<0.05	yes	yes	yes	yes	yes
[86]	Human blood lymphocytes	x		847.74 MHz	4.9	24 h			x	no	2	<0.05	yes	yes	yes	no	yes
	Human blood lymphocytes	x		847.74 MHz	5.5	24 h			x	no	2	<0.05	yes	yes	yes	no	yes
[87]	Human blood lymphocytes	x		835.62 MHz	4.4	24 h			x	no	2	<0.05	yes	yes	yes	no	yes
	Human blood lymphocytes	x		835.62 MHz	5	24 h			x	no	2	<0.05	yes	yes	yes	no	yes
	Human blood lymphocytes	x		2450 MHz	2.135	2 h			x	no	3	<0.05	yes	yes	yes	no	yes
	Human blood lymphocytes	x		8.2 GHz	20.71	2 h			x	no	2	<0.05	yes	yes	yes	no	yes
[88]	Human blood lymphocytes	x		2450 MHz	10.9	2 h			x	no	4	<0.05	no	no	no	no	yes
	Human blood lymphocytes	x		2451 MHz	10.9	2 h			x	no	4	<0.05	yes	yes	yes	yes	yes
[89]	Human blood lymphocytes	x		1800 MHz	0.2	28 h			x	no	20	<0.05	yes	yes	yes	yes	yes
	Human blood lymphocytes	x		1800 MHz	2	28 h			x	no	20	<0.05	yes	yes	yes	yes	yes
	Human blood lymphocytes	x		1800 MHz	10	28 h			x	no	20	<0.05	yes	yes	yes	yes	yes
[90]	Rat Sertoli cells	x		3 GHz		4 min	x	yes			3	<0.05	yes	no	no	no	no
[91]	Chinese hamster lung cells (CHLs)		x	1800 MHz	3	1 h			x	no	4	<0.05	yes	yes	yes	yes	yes
	Chinese hamster lung cells (CHLs)		x	1800 MHz	3	24 h			x	no	4	<0.05	yes	yes	yes	yes	yes
	Primary rat astrocytes	x		1800 MHz	3	1 h			x	no	4	<0.05	yes	yes	yes	yes	yes
	Primary rat astrocytes	x		1800 MHz	3	24 h			x	no	4	<0.05	yes	yes	yes	yes	yes
	Human amniotic epithelial cells (FLS)	x		1800 MHz	3	1 h			x	no	4	<0.05	yes	yes	yes	yes	yes

	Human amniotic epithelial cells (FLS)	x		1800 MHz		3	24 h			x	no	4	<0.05	yes	yes	yes	yes	yes
	Human lens epithelial cells (HLECs)	x		1800 MHz		3	1 h			x	no	4	<0.05	yes	yes	yes	yes	yes
	Human lens epithelial cells (HLECs)	x		1800 MHz		3	24 h			x	no	4	<0.05	yes	yes	yes	yes	yes
	Human skin fibroblasts (HSFs)		x	1800 MHz		3	1 h			x	no	4	<0.05	yes	yes	yes	yes	yes
	Human skin fibroblasts (HSFs)		x	1800 MHz		3	24 h			x	no	4	<0.05	yes	yes	yes	yes	yes
	Human umbilical vein endothelial cells (HUVECs)		x	1800 MHz		3	1 h			x	no	4	<0.05	yes	yes	yes	yes	yes
	Human umbilical vein endothelial cells (HUVECs)		x	1800 MHz		3	24 h			x	no	4	<0.05	yes	yes	yes	yes	yes
[92]	NIH3T3 cells (mouse fibroblasts)		x	916 MHz	10 W/m²		Intermittent			x	yes	n.s.	no	no	no	no	no	no
	NIH3T3 cells (mouse fibroblasts)		x	916 MHz	50 W/m²		Intermittent			x	yes	n.s.	no	no	no	no	no	no
	NIH3T3 cells (mouse fibroblasts)		x	916 MHz	90 W/m²		Intermittent			x	yes	n.s.	no	no	no	no	no	no
[93]	Rabbit lens epithelial cells (RLEC)	x		2.450 MHz	0.10 mW/cm².		8 h			x	no	n.s.	<0.01	yes	no	no	no	yes
	Rabbit lens epithelial cells (RLEC)	x		2.450 MHz	0.25 mW/cm².		8 h			x	no	n.s.	<0.01	yes	no	no	no	yes
	Rabbit lens epithelial cells (RLEC)	x		2.450 MHz	0.50 mW/cm².		8 h			x	yes	n.s.	<0.01	yes	no	no	no	yes
	Rabbit lens epithelial cells (RLEC)	x		2.450 MHz	1.00 mW/cm².		8 h			x	yes	n.s.	<0.01	yes	no	no	no	yes
	Rabbit lens epithelial cells (RLEC)	x		2.450 MHz	2.00 mW/cm2.		8 h			x	yes	n.s.	<0.01	yes	no	no	no	yes
[94]	Hdpc (human dermal papilla cells, NIH3T3 cells)		x	1763 MHz		10	1 h	x	yes			3	<0.05	yes	yes	no	no	yes
	Hair matrix keratinocytes	x		1763 MHz		10	Intermittent	x	yes			3	<0.05	yes	yes	no	no	yes
	Hdpc (human dermal papilla cells, NIH3T3 cells)		x	1763 MHz		10	1 h			x	yes	3	<0.05	yes	yes	no	no	yes
	Hair matrix keratinocytes	x		1763 MHz		10	Intermittent			x	yes	3	<0.05	yes	yes	no	no	yes
[95]	Human lymphocytes	x		900 MHz		0.2	intermittent			x	no	4-6 donors	<0.05	no	yes	no	no	yes
	Human lymphocytes	x		900 MHz		1.6	intermittent			x	no	4-6 donors	<0.05	no	yes	no	no	yes
	Human lymphocytes	x		900 MHz		1.6	intermittent			x	no	4-6 donors	<0.05	no	yes	no	no	yes
	Human lymphocytes	x		900 MHz		1.6	intermittent			x	no	4-6 donors	<0.05	no	yes	no	no	yes
[96]	Human lymphocytes	x		900 MHz		0.3	2 h			x	no	4-5 donors	<0.05	yes	yes	yes	no	Yes
	Human lymphocytes	x		900 MHz		1	2 h			x	no	4-5 donors	<0.05	yes	yes	yes	no	Yes
[97]	Human lymphocytes	x		120 GHz		1	20 min			x	no	3-4 donors	<0.05	yes	yes	no	yes	yes
	Human lymphocytes	x		130 GHz		0.6	20 min			x	no	3-4 donors	<0.05	yes	yes	no	yes	yes
	Human lymphocytes	x		130 GHz		3.5	20 min			x	no	3-4 donors	<0.05	yes	yes	no	yes	yes
	Human lymphocytes	x		130 GHz		5	20 min			x	no	3-4 donors	<0.05	yes	yes	no	yes	yes
[98]	Human lymphocytes	x		1950 MHz		2.2	Intermittent			x	no	6	<0.05	yes	yes	yes	yes	yes
	Human lymphocytes	x		1950 MHz		2.2	Intermittent			x	no	6	<0.05	yes	yes	yes	yes	yes

	Human lymphocytes	x		1950 MHz		2.2	Intermittent			x	no	6	<0.05	yes	yes	yes	yes	yes
[99]	Human lymphocytes	x		1950 MHz		1.25	20 h			x	no	3	<0.001	yes	yes	yes	yes	yes
	Human lymphocytes	x		1950 MHz		0.6	20 h			x	no	3	<0.001	yes	yes	yes	yes	yes
	Human lymphocytes	x		1950 MHz		0.3	20 h			x	no	3	<0.001	yes	yes	yes	yes	yes
	Human lymphocytes	x		1950 MHz		0.15	20 h			x	no	3	<0.001	yes	yes	yes	yes	yes
[100]	PC-12 cells		x	1950 MHz		10	24 h	x	no			3	<0.05	yes	yes	yes	yes	yes
	PC-12 cells		x	1950 MHz		10	24 h	x	no			3	<0.05	yes	yes	yes	yes	yes
[101]	Human B lymphoblastoid cells		x	1800 MHz		2	Intermittent	x	yes			3	<0.05	yes	yes	yes	no	yes
	Human B lymphoblastoid cells		x	1800 MHz		2	Intermittent			x	yes	3	<0.05	yes	yes	yes	no	yes
[102]	Pc12		x	2856 MHz	10 mW/cm²		5 min	x	no			3	<0.05	yes	no	no	yes	yes
	Pc12		x	2856 MHz	30 mW/cm²		5 min	x	yes			3	<0.01	yes	no	no	yes	yes
	Pc12		x	2856 MHz	50 mW/cm²		5 min	x	yes			3	<0.05	yes	no	no	yes	yes
	Pc12		x	2856 MHz	100 mW/cm²		5 min	x	yes			3	<0.01	yes	no	no	yes	yes
[103]	Human lymphocytes	x		2450 MHz	30 mW/cm²		15 min			x	no	2	<0.05	no	no	no	yes	no
	Human lymphocytes	x		2450 MHz	30 mW/cm²		30 min			x	no	2	<0.05	no	no	no	yes	no
	Human lymphocytes	x		2450 MHz	30 mW/cm²		60 min			x	no	2	<0.05	no	no	no	yes	no
	Human lymphocytes	x		7700 MHz	30 mW/cm²		15 min			x	no	2	<0.05	no	no	no	yes	no
	Human lymphocytes	x		7700 MHz	30 mW/cm²		30 min			x	no	2	<0.05	no	no	no	yes	no
	Human lymphocytes	x		7700 MHz	30 mW/cm²		60 min			x	no	2	<0.05	no	no	no	yes	no
[104]	Human lymphocytes	x		1800 MHz	5 mW/cm²		60 min			x	no	9	<0.05	no	no	no	no	no
	Human lymphocytes	x		1800 MHz	5 mW/cm²		120 min			x	no	9	<0.05	no	no	no	no	no
	Human lymphocytes	x		1800 MHz	5 mW/cm²		180 min			x	no	9	<0.05	no	no	no	no	no
	Human lymphocytes	x		1800 MHz	10 mW/cm²		60 min			x	no	9	<0.05	no	no	no	no	no
	Human lymphocytes	x		1800 MHz	10 mW/cm²		120 min			x	no	9	<0.05	no	no	no	no	no
	Human lymphocytes	x		1800 MHz	10 mW/cm²		180 min			x	no	9	<0.05	no	no	no	no	no
	Human lymphocytes	x		1800 MHz	20 mW/cm²		60 min			x	no	9	<0.05	no	no	no	no	no
	Human lymphocytes	x		1800 MHz	20 mW/cm²		120 min			x	no	9	<0.05	no	no	no	no	no
	Human lymphocytes	x		1800 MHz	20 mW/cm²		180 min			x	no	9	<0.05	no	no	no	no	no

n.s.: Not specified.

Table S2. Association test for exposure frequency, duration and SAR intervals.

Interval		IN (%) [a]	OUT (%) [b]	*p* Value
Frequency	≤1 GHz	22.9	20.8	0.32
	1–3 GHz	21.5	21.9	0.49
	>3 GHz	15.6	22.1	0.26
Exposure duration	≤1 h	24.7	21.1	0.29
	1–24 h	22.9	20.4	0.29
	>24 h	18.3	23.3	0.12
SAR	≤1 W/kg	22.3	19.1	0.24
	1–2 W/kg	19.2	21.1	0.41
	>2 W/kg	19.0	21.5	0.33

[a] IN column shows the ratio of positive findings inside the corresponding interval; [b] OUT column shows the ratio outside the defined interval.

References

1. Antonopoulos, A.; Eisenbrandt, H.; Obe, G. Effects of high-frequency electromagnetic fields on human lymphocytes in vitro. *Mutat. Res.* **1997**, *395*, 209–214.
2. Atasoy, A.; Sevim, Y.; Kaya, I.; Yilmaz, M.; Durmus, A.; Sonmez, M.; Omay, S.B.; Ozdemir, F.; Ovali, E. The effects of electromagnetic fields on peripheral blood mononuclear cells in vitro. *Bratisl. Lek. Listy.* **2009**, *110*, 526–529.
3. Avendano, C.; Mata, A.; Sanchez Sarmiento, C.A.; Doncel, G.F. Use of laptop computers connected to internet through wi-fi decreases human sperm motility and increases sperm DNA fragmentation. *Fertil. Steril.* **2012**, doi:10.1016/j.fertnstert.2011.10.012.
4. Belyaev, I.Y.; Hillert, L.; Protopopova, M.; Tamm, C.; Malmgren, L.O.; Persson, B.R.; Selivanova, G.; Harms-Ringdahl, M. 915 MHz microwaves and 50 Hz magnetic field affect chromatin conformation and 53BP1 foci in human lymphocytes from hypersensitive and healthy persons. *Bioelectromagnetics* **2005**, *26*, 173–184.
5. Beneduci, A.; Chidichimo, G.; De Rose, R.; Filippelli, L.; Straface, S.V.; Venuta, S. Frequency and irradiation time-dependant antiproliferative effect of low-power millimeter waves on RPMI 7932 human melanoma cell line. *Anticancer Res.* **2005**, *25*, 1023–1028.
6. Beneduci, A.; Chidichimo, G.; Tripepi, S.; Perrotta, E.; Cufone, F. Antiproliferative effect of millimeter radiation on human erythromyeloid leukemia cell line K562 in culture: Ultrastructural- and metabolic-induced changes. *Bioelectrochemistry* **2007**, *70*, 214–220.
7. Beneduci, A. Evaluation of the potential in vitro antiproliferative effects of millimeter waves at some therapeutic frequencies on RPMI 7932 human skin malignant melanoma cells. *Cell. Biochem. Biophys.* **2009**, *55*, 25–32.
8. Bock, J.; Fukuyo, Y.; Kang, S.; Phipps, M.L.; Alexandrov, L.B.; Rasmussen, K.O.; Bishop, A.R.; Rosen, E.D.; Martinez, J.S.; Chen, H.T.; et al. Mammalian stem cells reprogramming in response to terahertz radiation. *PLoS ONE* **2010**, doi:10.1371/journal.pone.0015806.
9. Bourthoumieu, S.; Magnaudeix, A.; Terro, F.; Leveque, P.; Collin, A.; Yardin, C. Study of P53 expression and post-transcriptional modifications after GSM—900 Radiofrequency exposure of human amniotic cells. *Bioelectromagnetics* **2013**, *34*, 52–60.
10. Buttiglione, M.; Roca, L.; Montemurno, E.; Vitiello, F.; Capozzi, V.; Cibelli, G. Radiofrequency radiation (900 MHz) induces Egr-1 gene expression and affects cell-cycle control in human neuroblastoma cells. *J. Cell. Physiol.* **2007**, *213*, 759–767.
11. Calabro, E.; Condello, S.; Curro, M.; Ferlazzo, N.; Caccamo, D.; Magazu, S.; Ientile, R. Modulation of heat shock protein response in SH-SY5Y by mobile phone microwaves. *World J. Biol. Chem.* **2012**, *3*, 34–40.
12. Canseven, A.G.; Esmekaya, M.A.; Kayhan, H.; Tuysuz, M.Z.; Seyhan, N. Effects of microwave exposure and Gemcitabine treatment on apoptotic activity in Burkitt's lymphoma (Raji) cells. *Electromagn. Biol. Med.* **2015**, 1–5.

13. Cao, Y.; Zhang, W.; Lu, M.X.; Xu, Q.; Meng, Q.Q.; Nie, J.H.; Tong, J. 900-MHz microwave radiation enhances gamma-ray adverse effects on SHG44 cells. *J. Toxicol. Environ. Health A* **2009**, *72*, 727–732.
14. Capri, M.; Scarcella, E.; Fumelli, C.; Bianchi, E.; Salvioli, S.; Mesirca, P.; Agostini, C.; Antolini, A.; Schiavoni, A.; Castellani, G.; et al. In vitro exposure of human lymphocytes to 900 MHz CW and GSM modulated radiofrequency: Studies of proliferation, apoptosis and mitochondrial membrane potential. *Radiat. Res.* **2004**, *162*, 211–218.
15. Caraglia, M.; Marra, M.; Mancinelli, F.; D'Ambrosio, G.; Massa, R.; Giordano, A.; Budillon, A.; Abbruzzese, A.; Bismuto, E. Electromagnetic fields at mobile phone frequency induce apoptosis and inactivation of the multi-chaperone complex in human epidermoid cancer cells. *J. Cell. Physiol.* **2005**, *204*, 539–548.
16. Chauhan, V.; Mariampillai, A.; Kutzner, B.C.; Wilkins, R.C.; Ferrarotto, C.; Bellier, P.V.; Marro, L.; Gajda, G.B.; Lemay, E.; Thansandote, A.; et al. Evaluating the biological effects of intermittent 1.9 GHz pulse-modulated radiofrequency fields in a series of human-derived cell lines. *Radiat. Res.* **2007**, *167*, 87–93.
17. Chen, C.; Ma, Q.; Liu, C.; Deng, P.; Zhu, G.; Zhang, L.; He, M.; Lu, Y.; Duan, W.; Pei, L.; et al. Exposure to 1800 MHz radiofrequency radiation impairs neurite outgrowth of embryonic neural stem cells. *Sci. Rep.* **2014**, *4*, 5103.
18. Cleary, S.F.; Cao, G.; Liu, L.M. Effects of isothermal 2.45 GHz microwave radiation on the mammalian cell cycle: Comparison with effects of isothermal 27 MHz radiofrequency radiation exposure. *Bioelectrochem. Bioenerg.* **1996**, *39*, 167–173.
19. Czyz, J.; Guan, K.; Zeng, Q.; Nikolova, T.; Meister, A.; Schonborn, F.; Schuderer, J.; Kuster, N.; Wobus, A.M. High frequency electromagnetic fields (GSM signals) affect gene expression levels in tumor suppressor P53-deficient embryonic stem cells. *Bioelectromagnetics* **2004**, *25*, 296–307.
20. Del Vecchio, G.; Giuliani, A.; Fernandez, M.; Mesirca, P.; Bersani, F.; Pinto, R.; Ardoino, L.; Lovisolo, G.A.; Giardino, L.; Calza, L. Effect of radiofrequency electromagnetic field exposure on *in vitro* models of neurodegenerative disease. *Bioelectromagnetics* **2009**, *30*, 564–572.
21. Duranti, G.; Rossi, A.; Rosato, N.; Fazio, G.; Sacerdoti, G.; Rossi, P.; Falsaperla, R.; Cannelli, V.; Supino, R. In vitro evaluation of biological effects on human keratinocytes exposed to 900 MHz electromagnetic field. *Environmentalist* **2005**, *25*, 113–119.
22. Esmekaya, M.A.; Aytekin, E.; Ozgur, E.; Guler, G.; Ergun, M.A.; Omeroglu, S.; Seyhan, N. Mutagenic and morphologic impacts of 1.8 GHz radiofrequency radiation on human peripheral blood lymphocytes (hPBLs) and possible protective role of pre-treatment with ginkgo biloba (EGB 761). *Sci. Total Environ.* **2011**, *410–411*, 59–64.
23. Falzone, N.; Huyser, C.; Franken, D.R.; Leszczynski, D. Mobile phone radiation does not induce pro-apoptosis effects in human spermatozoa. *Radiat. Res.* **2010**, *174*, 169–176.
24. French, P.; Donnellan, M.; McKenzie, D.R. Electromagnetic radiation at 835 MHz changes the morphology and inhibits proliferation of a human astrocytoma cell line. *Bioelectrochem. Bioenerg.* **1997**, *43*, 13–18.
25. Gurisik, E.; Warton, K.; Martin, D.K.; Valenzuela, S.M. An in vitro study of the effects of exposure to a GSM signal in two human cell lines: Monocytic U937 and neuroblastoma SK-N-SH. *Cell. Biol. Int.* **2006**, *30*, 793–799.
26. Higashikubo, R.; Ragouzis, M.; Moros, E.G.; Straube, W.L.; Roti Roti, J.L. Radiofrequency electromagnetic fields do not alter the cell cycle progression of C3H 10T and U87MG cells. *Radiat. Res.* **2001**, *156*, 786–795.
27. Hintzsche, H.; Jastrow, C.; Kleine-Ostmann, T.; Schrader, T.; Stopper, H. 900 MHz radiation does not induce micronucleus formation in different cell types. *Mutagenesis* **2012**, *27*, 477–483.
28. Hintzsche, H.; Jastrow, C.; Kleine-Ostmann, T.; Karst, U.; Schrader, T.; Stopper, H. Terahertz electromagnetic fields (0.106 THz) do not induce manifest genomic damage in vitro. *PLoS ONE* **2012**, doi:10.1371/journal.pone.0046397.
29. Hirose, H.; Sakuma, N.; Kaji, N.; Suhara, T.; Sekijima, M.; Nojima, T.; Miyakoshi, J. Phosphorylation and gene expression of P53 are not affected in human cells exposed to 2.1425 GHz band Cw or W-CDMA modulated radiation allocated to mobile radio base stations. *Bioelectromagnetics* **2006**, *27*, 494–504.
30. Hoyto, A.; Sokura, M.; Juutilainen, J.; Naarala, J. Radiofrequency radiation does not significantly affect ornithine decarboxylase activity, proliferation, or caspase-3 activity of fibroblasts in different physiological conditions. *Int. J. Radiat. Biol.* **2008**, *84*, 727–733.
31. Hoyto, A.; Luukkonen, J.; Juutilainen, J.; Naarala, J. Proliferation, oxidative stress and cell death in cells exposed to 872 MHz radiofrequency radiation and oxidants. *Radiat. Res.* **2008**, *170*, 235–243.
32. Huang, T.Q.; Lee, M.S.; Oh, E.; Zhang, B.T.; Seo, J.S.; Park, W.Y. Molecular responses of Jurkat T-cells to 1763 MHz radiofrequency radiation. *Int. J. Radiat. Biol.* **2008**, *84*, 734–741.

33. Huang, T.Q.; Lee, M.S.; Oh, E.H.; Kalinec, F.; Zhang, B.T.; Seo, J.S.; Park, W.Y. Characterization of biological effect of 1763 MHz radiofrequency exposure on auditory hair cells. *Int. J. Radiat. Biol.* **2008**, *84*, 909–915.
34. Jin, Z.; Zong, C.; Jiang, B.; Zhou, Z.; Tong, J.; Cao, Y. The effect of combined exposure of 900 MHz radiofrequency fields and doxorubicin in HL-60 cells. *PLoS ONE* **2012**, doi:10.1371/journal.pone.0046102.
35. Joubert, V.; Leveque, P.; Rametti, A.; Collin, A.; Bourthoumieu, S.; Yardin, C. Microwave exposure of neuronal cells in vitro: Study of apoptosis. *Int. J. Radiat. Biol.* **2006**, *82*, 267–275.
36. Joubert, V.; Leveque, P.; Cueille, M.; Bourthoumieu, S.; Yardin, C. No apoptosis is induced in rat cortical neurons exposed to GSM phone fields. *Bioelectromagnetics* **2007**, *28*, 115–121.
37. Joubert, V.; Bourthoumieu, S.; Leveque, P.; Yardin, C. Apoptosis is induced by radiofrequency fields through the caspase-independent mitochondrial pathway in cortical neurons. *Radiat. Res.* **2008**, *169*, 38–45.
38. Kahya, M.C.; Naziroglu, M.; Cig, B. Selenium reduces mobile phone (900 MHz)—Induced oxidative stress, mitochondrial function, and apoptosis in breast cancer cells. *Biol. Trace Elem. Res.* **2014**, *160*, 285–293.
39. Karaca, E.; Durmaz, B.; Aktug, H.; Yildiz, T.; Guducu, C.; Irgi, M.; Koksal, M.G.; Ozkinay, F.; Gunduz, C.; Cogulu, O. The genotoxic effect of radiofrequency waves on mouse brain. *J. Neurooncol.* **2012**, *106*, 53–58.
40. Kumar, G.; Wood, A.W.; Anderson, V.; McIntosh, R.L.; Chen, Y.Y.; McKenzie, R.J. Evaluation of hematopoietic system effects after *in vitro* radiofrequency radiation exposure in rats. *Int. J. Radiat. Biol.* **2011**, *87*, 231–240.
41. Kwee, S.; Raskmark, P. Changes in cell proliferation due to environmental non-ionizing radiation: 2. Microwave radiation. *Bioelectrochem. Bioenerg.* **1998**, *44*, 251–255.
42. Lantow, M.; Viergutz, T.; Weiss, D.G.; Simko, M. Comparative study of cell cycle kinetics and induction of apoptosis or necrosis after exposure of human Mono Mac 6 cells to radiofrequency radiation. *Radiat. Res.* **2006**, *166*, 539–543.
43. Lee, J.J.; Kwak, H.J.; Lee, Y.M.; Lee, J.W.; Park, M.J.; Ko, Y.G.; Choi, H.D.; Kim, N.; Pack, J.K.; Hong, S.I.; et al. Acute radio frequency irradiation does not affect cell cycle, cellular migration, and invasion. *Bioelectromagnetics* **2008**, *29*, 615–625.
44. Lee, K.Y.; Kim, B.C.; Han, N.K.; Lee, Y.S.; Kim, T.; Yun, J.H.; Kim, N.; Pack, J.K.; Lee, J.S. Effects of combined radiofrequency radiation exposure on the cell cycle and its regulatory proteins. *Bioelectromagnetics* **2011**, *32*, 169–178.
45. Lee, S.; Johnson, D.; Dunbar, K.; Dong, H.; Ge, X.; Kim, Y.C.; Wing, C.; Jayathilaka, N.; Emmanuel, N.; Zhou, C.Q.; et al. 2.45 GHz radiofrequency fields alter gene expression in cultured human cells. *FEBS Lett.* **2005**, *579*, 4829–4836.
46. Lee, S.S.; Kim, H.R.; Kim, M.S.; Park, S.; Yoon, E.S.; Park, S.H.; Kim, D.W. Influence of smartphone Wi-Fi signals on adipose-derived stem cells. *J. Craniofac. Surg.* **2014**, *25*, 1902–1907.
47. Liu, K.; Zhang, G.; Wang, Z.; Liu, Y.; Dong, J.; Dong, X.; Liu, J.; Cao, J.; Ao, L.; Zhang, S. The protective effect of autophagy on mouse spermatocyte derived cells exposure to 1800 MHz radiofrequency electromagnetic radiation. *Toxicol. Lett.* **2014**, *228*, 216–224.
48. Liu, M.L.; Wen, J.Q.; Fan, Y.B. Potential protection of green tea polyphenols against 1800 MHz electromagnetic radiation-induced injury on rat cortical neurons. *Neurotoxic. Res.* **2011**, *20*, 270–276.
49. Liu, Y.X.; Tai, J.L.; Li, G.Q.; Zhang, Z.W.; Xue, J.H.; Liu, H.S.; Zhu, H.; Cheng, J.D.; Liu, Y.L.; Li, A.M.; et al. Exposure to 1950-MHz TD-SCDMA electromagnetic fields affects the apoptosis of astrocytes via caspase-3-dependent pathway. *PLoS One* **2012**, doi:org/10.1371/journal.pone.0042332.
50. Lixia, S.; Yao, K.; Kaijun, W.; Deqiang, L.; Huajun, H.; Xiangwei, G.; Baohong, W.; Wei, Z.; Jianling, L.; Wei, W. Effects of 1.8 GHz radiofrequency field on DNA damage and expression of heat shock protein 70 in human lens epithelial cells. *Mutat. Res.* **2006**, *602*, 135–142.
51. Lu, Y.S.; Huang, B.T.; Huang, Y.X. Reactive oxygen species formation and apoptosis in human peripheral blood mononuclear cell induced by 900 MHz mobile phone radiation. *Oxid. Med. Cell. Longevity* **2012**, doi:10.1155/2012/740280.
52. Maes, A.; Collier, M.; Slaets, D.; Verschaeve, L. 954 MHz microwaves enhance the mutagenic properties of mitomycin C. *Environ. Mol. Mutagen.* **1996**, *28*, 26–30.
53. Maioli, M.; Rinaldi, S.; Santaniello, S.; Castagna, A.; Pigliaru, G.; Gualini, S.; Cavallini, C.; Fontani, V.; Ventura, C. Radio electric conveyed fields directly reprogram human dermal skin fibroblasts toward cardiac, neuronal, and skeletal muscle-like lineages. *Cell. Transplant.* **2013**, *22*, 1227–1235.
54. Marinelli, F.; La Sala, D.; Cicciotti, G.; Cattini, L.; Trimarchi, C.; Putti, S.; Zamparelli, A.; Giuliani, L.; Tomassetti, G.; Cinti, C. Exposure to 900 MHz electromagnetic field induces an unbalance between

pro-apoptotic and pro-survival signals in T-lymphoblastoid leukemia CCRF-CEM cells. *J. Cell. Physiol.* **2004**, *198*, 324–332.

55. Merola, P.; Marino, C.; Lovisolo, G.A.; Pinto, R.; Laconi, C.; Negroni, A. Proliferation and apoptosis in a neuroblastoma cell line exposed to 900 MHz modulated radiofrequency field. *Bioelectromagnetics* **2006**, *27*, 164–171.
56. Miyakoshi, J.; Takemasa, K.; Takashima, Y.; Ding, G.R.; Hirose, H.; Koyama, S. Effects of exposure to a 1950 MHz radio frequency field on expression of Hsp70 and Hsp27 in human glioma cells. *Bioelectromagnetics* **2005**, *26*, 251–257.
57. Moisescu, M.G.; Leveque, P.; Bertrand, J.R.; Kovacs, E.; Mir, L.M. Microscopic observation of living cells during their exposure to modulated electromagnetic fields. *Bioelectrochemistry* **2008**, *74*, 9–15.
58. Moquet, J.; Ainsbury, E.; Bouffler, S.; Lloyd, D. Exposure to low level GSM 935 MHz radiofrequency fields does not induce apoptosis in proliferating or differentiated murine neuroblastoma cells. *Radiat. Prot. Dosimetry* **2008**, *131*, 287–296.
59. Naziroglu, M.; Cig, B.; Dogan, S.; Uguz, A.C.; Dilek, S.; Faouzi, D. 2.45-Gz wireless devices induce oxidative stress and proliferation through cytosolic ca^{2+} influx in human leukemia cancer cells. *Int. J. Radiat. Biol.* **2012**, *88*, 449–456.
60. Nikolova, T.; Czyz, J.; Rolletschek, A.; Blyszczuk, P.; Fuchs, J.; Jovtchev, G.; Schuderer, J.; Kuster, N.; Wobus, A.M. Electromagnetic fields affect transcript levels of apoptosis-related genes in embryonic stem cell-derived neural progenitor cells. *Faseb. J.* **2005**, *19*, 1686–1688.
61. Ozgur, E.; Guler, G.; Kismali, G.; Seyhan, N. Mobile phone radiation alters proliferation of hepatocarcinoma cells. *Cell. Biochem. Biophys.* **2014**, *70*, 983–991.
62. Pacini, S.; Ruggiero, M.; Sardi, I.; Aterini, S.; Gulisano, F.; Gulisano, M. Exposure to global system for mobile communication (GSM) cellular phone radiofrequency alters gene expression, proliferation, and morphology of human skin fibroblasts. *Oncol. Res.* **2002**, *13*, 19–24.
63. Palumbo, R.; Brescia, F.; Capasso, D.; Sannino, A.; Sarti, M.; Capri, M.; Grassilli, E.; Scarfi, M.R. Exposure to 900 MHz radiofrequency radiation induces caspase 3 activation in proliferating human lymphocytes. *Radiat. Res.* **2008**, *170*, 327–334.
64. Pavicic, I.; Trosic, I. Influence of 864 MHz electromagnetic field on growth kinetics of established cell line. *Biologia* **2006**, *61*, 321–325.
65. Pavicic, I.; Trosic, I. In vitro testing of cellular response to ultra high frequency electromagnetic field radiation. *Toxicol. In Vitro* **2008**, *22*, 1344–1348.
66. Pavicic, I.; Trosic, I. Impact of 864 Mhz or 935 MHz radiofrequency microwave radiation on the basic growth parameters of V79 cell line. *Acta Biol. Hung.* **2008**, *59*, 67–76.
67. Peinnequin, A.; Piriou, A.; Mathieu, J.; Dabouis, V.; Sebbah, C.; Malabiau, R.; Debouzy, J.C. Non-thermal effects of continuous 2.45 GHz microwaves on fas-induced apoptosis in human jurkat T-cell line. *Bioelectrochemistry* **2000**, *51*, 157–161.
68. Perez-Castejon, C.; Perez-Bruzon, R.N.; Llorente, M.; Pes, N.; Lacasa, C.; Figols, T.; Lahoz, M.; Maestu, C.; Vera-Gil, A.; Del Moral, A.; et al. Exposure to elf-pulse modulated x band microwaves increases *in vitro* human astrocytoma cell proliferation. *Histol. Histopathol.* **2009**, *24*, 1551–1561.
69. Port, M.; Abend, M.; Romer, B.; Van Beuningen, D. Influence of high-frequency electromagnetic fields on different modes of cell death and gene expression. *Int. J. Radiat. Biol.* **2003**, *79*, 701–708.
70. Sanchez, S.; Milochau, A.; Ruffie, G.; Poulletier de Gannes, F.; Lagroye, I.; Haro, E.; Surleve-Bazeille, J.E.; Billaudel, B.; Lassegues, M.; Veyret, B. Human skin cell stress response to GSM—900 Mobile phone signals. *In vitro* study on isolated primary cells and reconstructed epidermis. *FEBS J.* **2006**, *273*, 5491–5507.
71. Sannino, A.; Zeni, O.; Romeo, S.; Massa, R.; Gialanella, G.; Grossi, G.; Manti, L.; Vijayalaxmi; Scarfi, M.R. Adaptive response in human blood lymphocytes exposed to non-ionizing radiofrequency fields: Resistance to ionizing radiation-induced damage. *J. Radiat. Res.* **2014**, *55*, 210–217.
72. Sannino, A.; Zeni, O.; Sarti, M.; Romeo, S.; Reddy, S.B.; Belisario, M.A.; Prihoda, T.J.; Vijayalaxmi; Scarfi, M.R. Induction of adaptive response in human blood lymphocytes exposed to 900 MHz radiofrequency fields: Influence of cell cycle. *Int. J. Radiat. Biol.* **2011**, *87*, 993–999.
73. Sannino, A.; Sarti, M.; Reddy, S.B.; Prihoda, T.J.; Vijayalaxmi; Scarfi, M.R. Induction of adaptive response in human blood lymphocytes exposed to radiofrequency radiation. *Radiat. Res.* **2009**, *171*, 735–742.

74. Sannino, A.; Di Costanzo, G.; Brescia, F.; Sarti, M.; Zeni, O.; Juutilainen, J.; Scarfi, M.R. Human fibroblasts and 900 MHz radiofrequency radiation: Evaluation of DNA damage after exposure and co-exposure to 3-chloro-4-(dichloromethyl)-5-hydroxy-2(5h)-furanone (MX). *Radiat. Res.* **2009**, *171*, 743–751.
75. Scarfi, M.R.; Fresegna, A.M.; Villani, P.; Pinto, R.; Marino, C.; Sarti, M.; Altavista, P.; Sannino, A.; Lovisolo, G.A. Exposure to radiofrequency radiation (900 MHz, GSM signal) does not affect micronucleus frequency and cell proliferation in human peripheral blood lymphocytes: An interlaboratory study. *Radiat. Res.* **2006**, *165*, 655–663.
76. Schrader, T.; Munter, K.; Kleine-Ostmann, T.; Schmid, E. Spindle disturbances in human-hamster hybrid (AL) cells induced by mobile communication frequency range signals. *Bioelectromagnetics* **2008**, *29*, 626–639.
77. Schrader, T.; Kleine-Ostmann, T.; Munter, K.; Jastrow, C.; Schmid, E. Spindle disturbances in human-hamster hybrid (AL) cells induced by the electrical component of the mobile communication frequency range signal. *Bioelectromagnetics* **2011**, *32*, 291–301.
78. Sekijima, M.; Takeda, H.; Yasunaga, K.; Sakuma, N.; Hirose, H.; Nojima, T.; Miyakoshi, J. 2-GHz band CW and W-CDMA modulated radiofrequency fields have no significant effect on cell proliferation and gene expression profile in human cells. *J. Radiat. Res.* **2010**, *51*, 277–284.
79. Stagg, R.B.; Thomas, W.J.; Jones, R.A.; Adey, W.R. DNA synthesis and cell proliferation in C6 glioma and primary glial cells exposed to a 836.55 MHz modulated radiofrequency field. *Bioelectromagnetics* **1997**, *18*, 230–236.
80. Szabo, I.; Rojavin, M.A.; Rogers, T.J.; Ziskin, M.C. Reactions of keratinocytes to *in vitro* millimeter wave exposure. *Bioelectromagnetics* **2001**, *22*, 358–364.
81. Takashima, Y.; Hirose, H.; Koyama, S.; Suzuki, Y.; Taki, M.; Miyakoshi, J. Effects of continuous and intermittent exposure to RF fields with a wide range of SARs on cell growth, survival, and cell cycle distribution. *Bioelectromagnetics* **2006**, *27*, 392–400.
82. Terro, F.; Magnaudeix, A.; Crochetet, M.; Martin, L.; Bourthoumieu, S.; Wilson, C.M.; Yardin, C.; Leveque, P. GSM-900 MHz at low dose temperature-dependently downregulates alpha-synuclein in cultured cerebral cells independently of chaperone-mediated-autophagy. *Toxicology* **2012**, *292*, 136–144.
83. Trillo, M.A.; Cid, M.A.; Martinez, M.A.; Page, J.E.; Esteban, J.; Ubeda, A. Cytostatic response of NB69 cells to weak pulse-modulated 2.2 GHz radar-like signals. *Bioelectromagnetics* **2011**, *32*, 340–350.
84. Velizarov, S.; Raskmark, P.; Kwee, S. The effects of radiofrequency fields on cell proliferation are non-thermal. *Bioelectrochem. Bioenerg.* **1999**, *48*, 177–180.
85. Vijayalaxmi; Mohan, N.; Meltz, M.L.; Wittler, M.A. Proliferation and cytogenetic studies in human blood lymphocytes exposed *in vitro* to 2450 MHz radiofrequency radiation. *Int. J. Radiat. Biol.* **1997**, *72*, 751–757.
86. Vijayalaxmi; Bisht, K.S.; Pickard, W.F.; Meltz, M.L.; Roti Roti, J.L.; Moros, E.G. Chromosome damage and micronucleus formation in human blood lymphocytes exposed in vitro to radiofrequency radiation at a cellular telephone frequency (847.74 MHz, CDMA). *Radiat. Res.* **2001**, *156*, 430–432.
87. Vijayalaxmi; Leal, B.Z.; Meltz, M.L.; Pickard, W.F.; Bisht, K.S.; Roti Roti, J.L.; Straube, W.L.; Moros, E.G. Cytogenetic studies in human blood lymphocytes exposed *in vitro* to radiofrequency radiation at a cellular telephone frequency (835.62 MHz, FDMA). *Radiat. Res.* **2001**, *155*, 113–121.
88. Vijayalaxmi; Reddy, A.B.; McKenzie, R.J.; McIntosh, R.L.; Prihoda, T.J.; Wood, A.W. Incidence of micronuclei in human peripheral blood lymphocytes exposed to modulated and unmodulated 2450 MHz radiofrequency fields. *Bioelectromagnetics* **2013**, *34*, 542–548.
89. Waldmann, P.; Bohnenberger, S.; Greinert, R.; Hermann-Then, B.; Heselich, A.; Klug, S.J.; Koenig, J.; Kuhr, K.; Kuster, N.; Merker, M.; et al. Influence of GSM signals on human peripheral lymphocytes: Study of genotoxicity. *Radiat. Res.* **2013**, *179*, 243–253.
90. Wu, H.; Wang, D.; Shu, Z.; Zhou, H.; Zuo, H.; Wang, S.; Li, Y.; Xu, X.; Li, N.; Peng, R. Cytokines produced by microwave-radiated sertoli cells interfere with spermatogenesis in rat testis. *Andrologia* **2012**, doi:10.1111/j.1439-0272.2011.01232.x.
91. Xu, S.; Chen, G.; Chen, C.; Sun, C.; Zhang, D.; Murbach, M.; Kuster, N.; Zeng, Q.; Xu, Z. Cell type-dependent induction of DNA damage by 1800 MHz radiofrequency electromagnetic fields does not result in significant cellular dysfunctions. *PLoS ONE* **2013**, doi:10.1371/journal.pone.0054906.
92. Yang, L.; Hao, D.; Wang, M.; Zeng, Y.; Wu, S.; Zeng, Y. Cellular neoplastic transformation induced by 916 MHz microwave radiation. *Cell. Mol. Neurobiol.* **2012**, *32*, 1039–1046.
93. Yao, K.; Wang, K.J.; Sun, Z.H.; Tan, J.; Xu, W.; Zhu, L.J.; Lu, D.Q. Low power microwave radiation inhibits the proliferation of rabbit lens epithelial cells by upregulating P27Kip1 expression. *Mol. Vis.* **2004**, *10*, 138–143.

94. Yoon, S.Y.; Kim, K.T.; Jo, S.J.; Cho, A.R.; Jeon, S.I.; Choi, H.D.; Kim, K.H.; Park, G.S.; Pack, J.K.; Kwon, O.S.; et al. Induction of hair growth by insulin-like growth factor-1 in 1763 MHz radiofrequency—Irradiated hair follicle cells. *PLoS ONE* **2011**, doi:10.1371/journal.pone.0028474.
95. Zeni, O.; Chiavoni, A.S.; Sannino, A.; Antolini, A.; Forigo, D.; Bersani, F.; Scarfi, M.R. Lack of genotoxic effects (micronucleus induction) in human lymphocytes exposed in vitro to 900 MHz electromagnetic fields. *Radiat. Res.* **2003**, *160*, 152–158.
96. Zeni, O.; Romano, M.; Perrotta, A.; Lioi, M.B.; Barbieri, R.; d'Ambrosio, G.; Massa, R.; Scarfi, M.R. Evaluation of genotoxic effects in human peripheral blood leukocytes following an acute in vitro exposure to 900 MHz radiofrequency fields. *Bioelectromagnetics* **2005**, *26*, 258–265.
97. Zeni, O.; Gallerano, G.P.; Perrotta, A.; Romano, M.; Sannino, A.; Sarti, M.; D'Arienzo, M.; Doria, A.; Giovenale, E.; Lai, A.; et al. Cytogenetic observations in human peripheral blood leukocytes following *in vitro* exposure to thz radiation: A pilot study. *Health Phys.* **2007**, *92*, 349–357.
98. Zeni, O.; Schiavoni, A.; Perrotta, A.; Forigo, D.; Deplano, M.; Scarfi, M.R. Evaluation of genotoxic effects in human leukocytes after in vitro exposure to 1950 MHz UMTS radiofrequency field. *Bioelectromagnetics* **2008**, *29*, 177–184.
99. Zeni, O.; Sannino, A.; Romeo, S.; Massa, R.; Sarti, M.; Reddy, A.B.; Prihoda, T.J.; Vijayalaxmi; Scarfi, M.R. Induction of an adaptive response in human blood lymphocytes exposed to radiofrequency fields: Influence of the universal mobile telecommunication system (UMTS) signal and the specific absorption rate. *Mutat. Res.* **2012**, *747*, 29–35.
100. Zeni,O.; Sannino,A.; Sarti,M.; Romeo,S.; Massa,R.; Scarfi,M.R. Radiofrequencyradiationat1950MHz(UMTS) does not affect key cellular endpoints in neuron—Like PC12 cells. *Bioelectromagnetics* **2012**, *33*, 497–507.
101. Zhijian, C.; Xiaoxue, L.; Wei, Z.; Yezhen, L.; Jianlin, L.; Deqiang, L.; Shijie, C.; Lifen, J.; Jiliang, H. Studying the protein expression in human B lymphoblastoid cells exposed to 1.8-GHz (GSM) radiofrequency radiation (RFR) with protein microarray. *Biochem. Biophys. Res. Commun.* **2013**, *433*, 36–39.
102. Zotti-Martelli, L.; Peccatori, M.; Scarpato, R.; Migliore, L. Induction of micronuclei in human lymphocytes exposed in vitro to microwave radiation. *Mutat. Res.* **2000**, *472*, 51–58.
103. Zotti-Martelli, L.; Peccatori, M.; Maggini, V.; Ballardin, M.; Barale, R. Individual responsiveness to induction of micronuclei in human lymphocytes after exposure in vitro to 1800-MHz microwave radiation. *Mutat. Res.* **2005**, *582*, 42–52.
104. Zuo, H.Y.; Lin, T.; Wang, D.W.; Peng, R.Y.; Wang, S.M.; Gao, Y.B.; Xu, X.P.; Li, Y.; Wang, S.X.; Zhao, L.; et al. Neural cell apoptosis induced by microwave exposure through mitochondria-dependent caspase-3 pathway. *Int. J. Med. Sci.* **2014**, *11*, 426–435.

© 2016 by the authors; licensee MDPI, Basel, Switzerland. This article is an open access article distributed under the terms and conditions of the Creative Commons by Attribution (CC-BY) license (http://creativecommons.org/licenses/by/4.0/).
